# Supplementary material for: A critical role of PRDM14 in human primordial germ cell fate revealed by inducible degrons
Source: Nat Commun. 2020 Mar 9;11:1282. doi: 10.1038/s41467-020-15042-0 (PMC7062732; doi:10.1038/s41467-020-15042-0)
Supplement: Supplementary file 1 — Supplementary Information [file 41467_2020_15042_MOESM1_ESM.pdf]

Supplementary information

A critical role of PRDM14 in human primordial germ cell fate revealed by inducible degrons

Sybirna *et al.*

Supplementary Figures

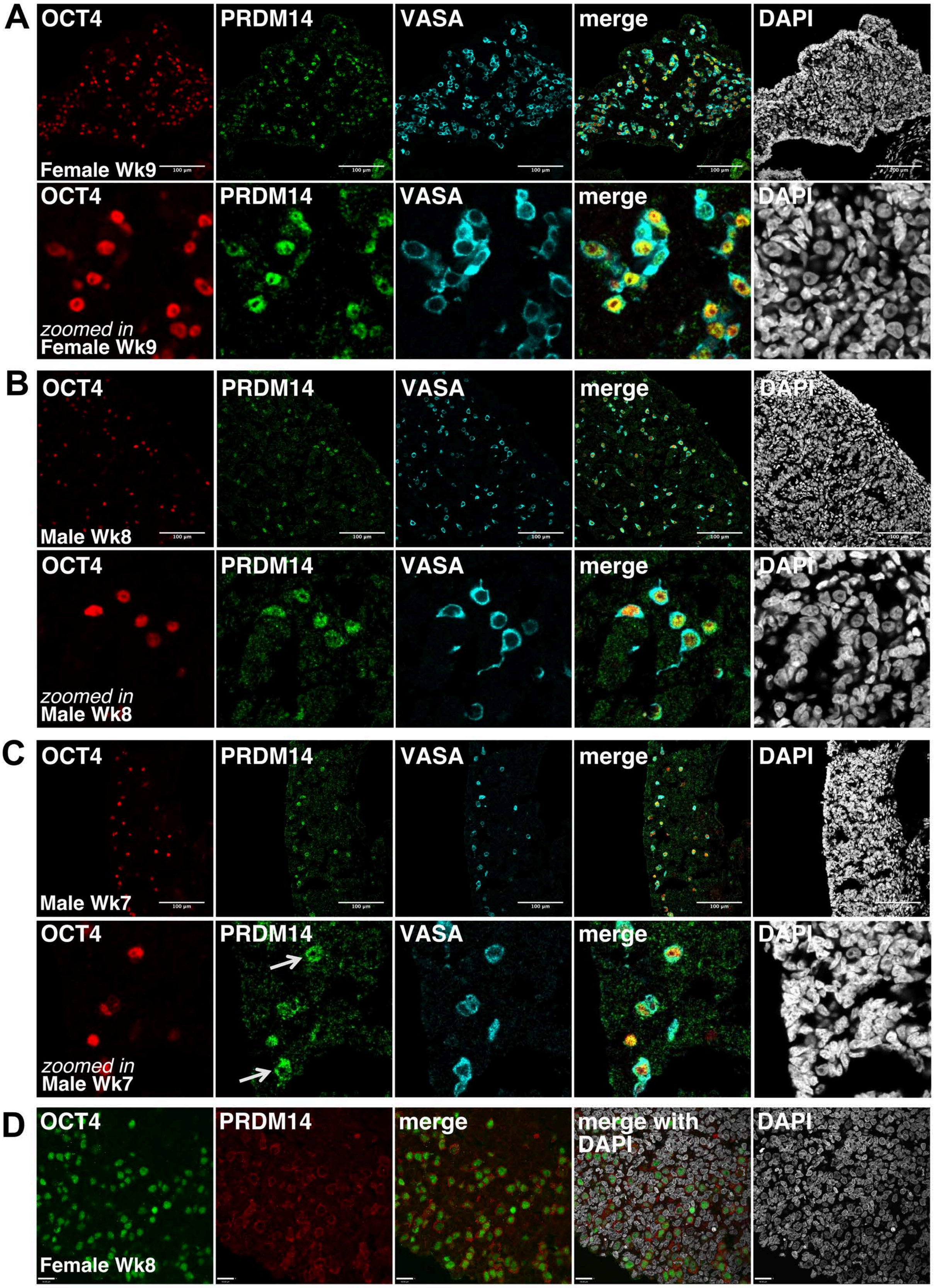

Supplementary Figure 1. Nuclear PRDM14 expression in human gonadal PGCs. (A-D) IF images of PRDM14 staining in human male and female gonadal sections of indicated stages (representative of 3 female and 2 male gonads). In A-C, hPGCs were marked by OCT4 (nuclear) and VASA (cytoplasmic) expression; in D, hPGCs are marked with OCT4 (nuclear) expression. Nuclei were counterstained by DAPI. Scale bar is 100 µm (A-C) or 60 µm (D). *(Supplementary Fig.1 is continued on the next page)*

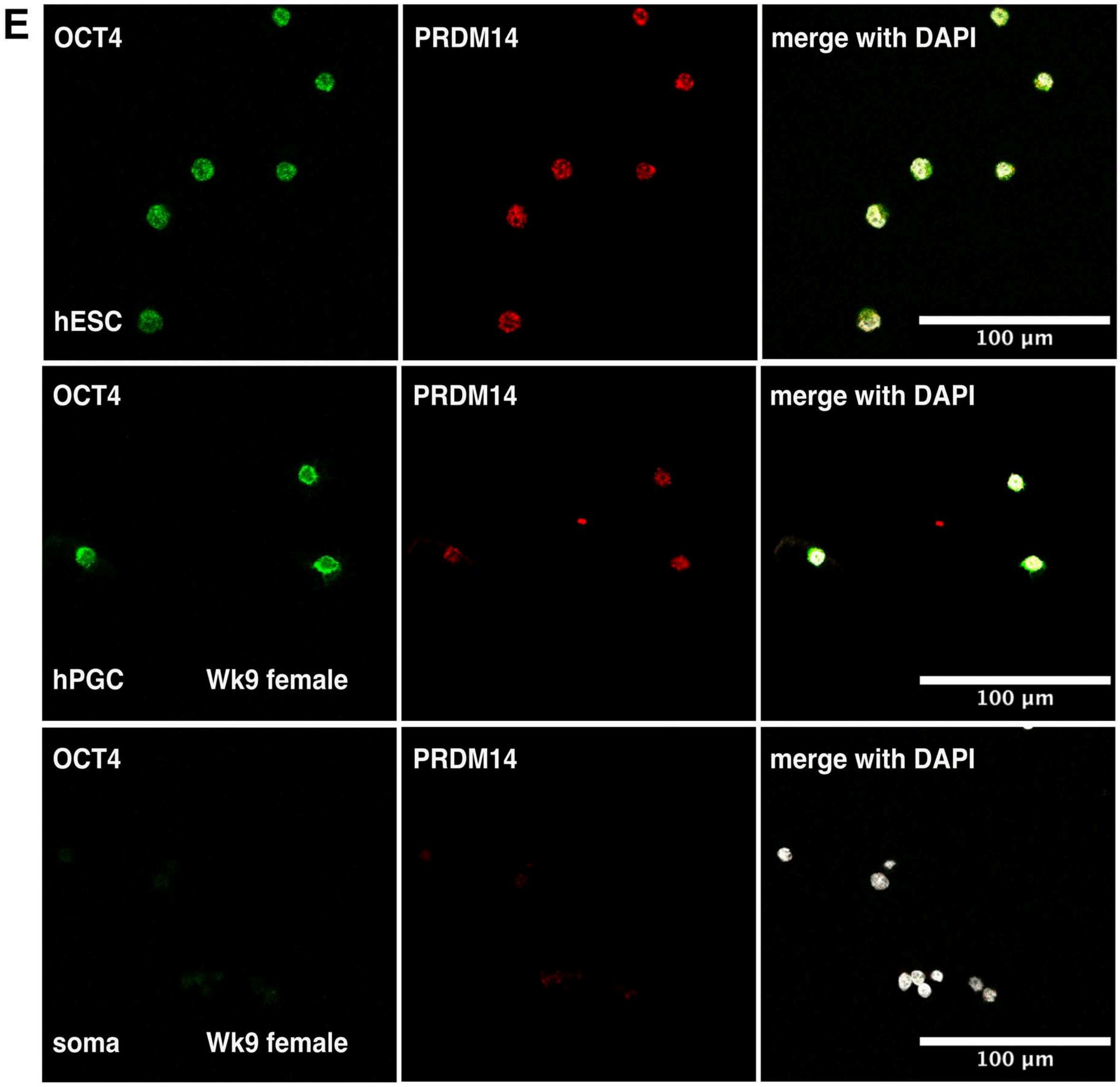

Supplementary Figure 1 (continued). Nuclear PRDM14 expression in human gonadal PGCs. (E) IF on sorted AP$^+$ hESCs and AP$^+$cKIT$^+$ gonadal hPGCs. hPGCs and pluripotent hESCs were marked by OCT4. Nuclei were counterstained by DAPI. AP$^-$cKIT$^-$ population (gonadal soma) was used as a negative control. Representative of 2 experiments (similar results were obtained for a Wk9 male embryo). Scale bar is 100 µm.

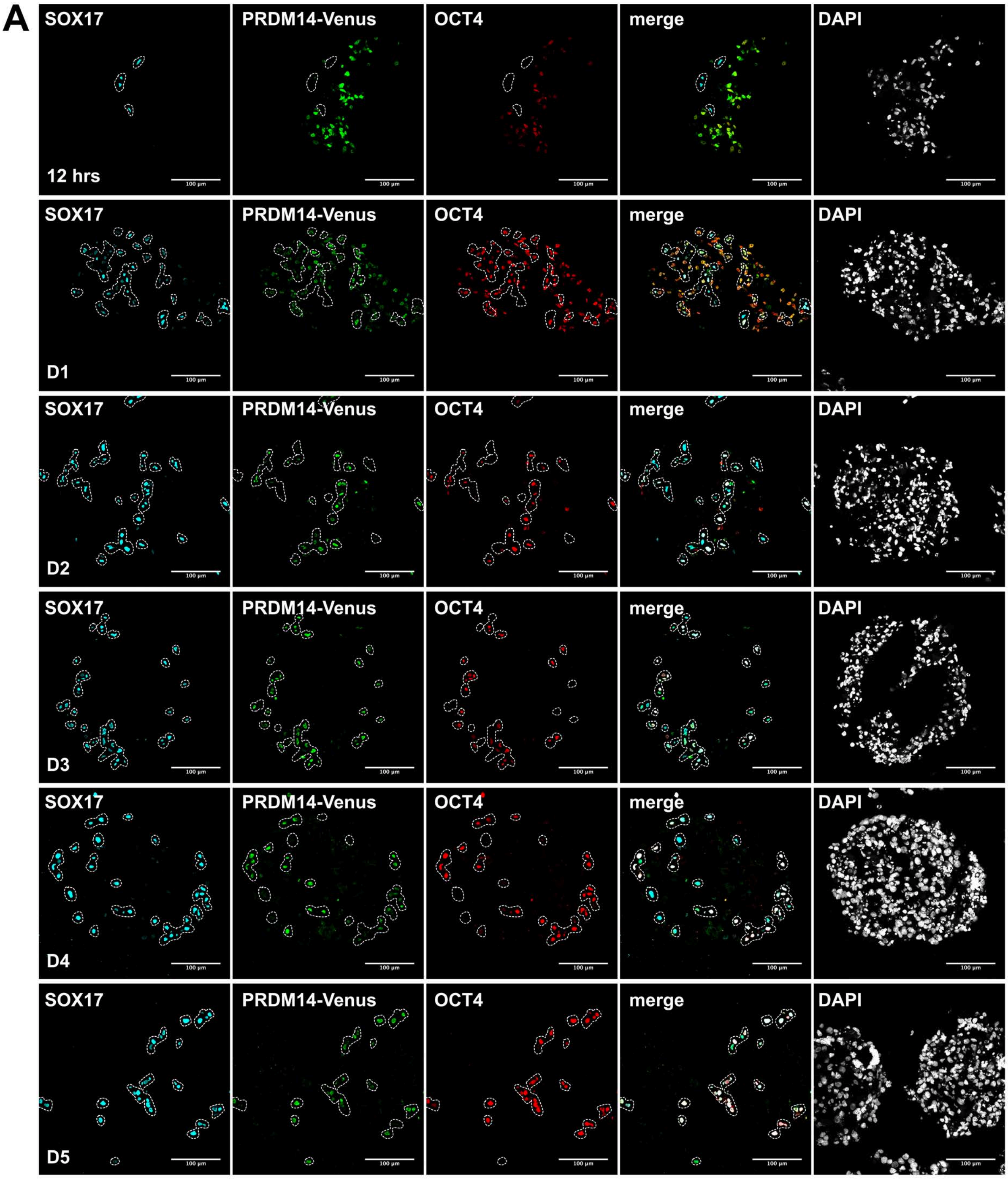

Supplementary Figure 2. Expression dynamics of PRDM14-Venus and other germ cell and pluripotency markers during hPGCLC specification. Time-course IF analysis showing indicated proteins' expression in embryoid body (EB) sections throughout hPGCLC specification from PRDM14-AID-Venus fusion reporter cell line. hPGCLCs were induced by cytokines (unless otherwise specified) and EBs were collected at 12hrs, and on D1-D5. Representative images for all timepoints are shown. Nuclei were counterstained by DAPI. Scale bar is 100 µm. **(A)** Time-course IF showing SOX17, PRDM14-Venus and OCT4 expression (representative of 2 experiments). Dashed line highlights SOX17^{+} cells. *(Supplementary Fig.2 is continued on the next page)*

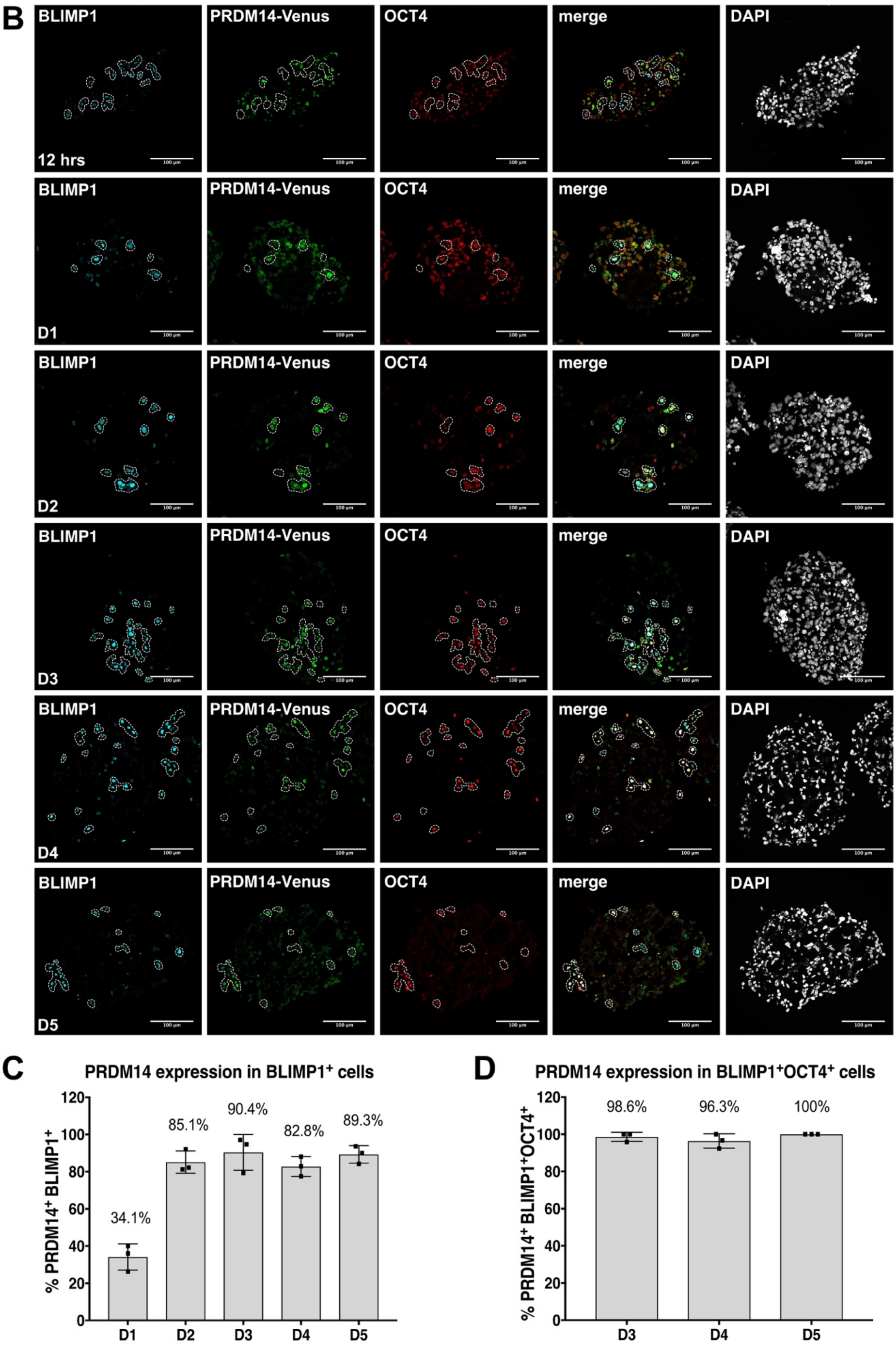

Supplementary Figure 2 (continued). Expression dynamics of PRDM14-Venus and other germ cell and pluripotency markers during hPGCLC specification. (B) Time-course IF showing BLIMP1, PRDM14-Venus and OCT4 expression (representative of 2 experiments). Dashed line highlights BLIMP1^{+} cells. **(C)** Quantification of results from (B) showing the percentage of PRDM14-Venus^{+}BLIMP1^{+} cells as mean+/-SD of n=3 EB sections from 1 hPGCLC induction. **(D)** Quantification of results from (B) showing the percentage of PRDM14-Venus^{+}BLIMP1^{+}OCT4^{+} cells as mean+/-SD of n=3 EB sections from 1 hPGCLC induction. *(Supplementary Fig2 is continued on the next page)*

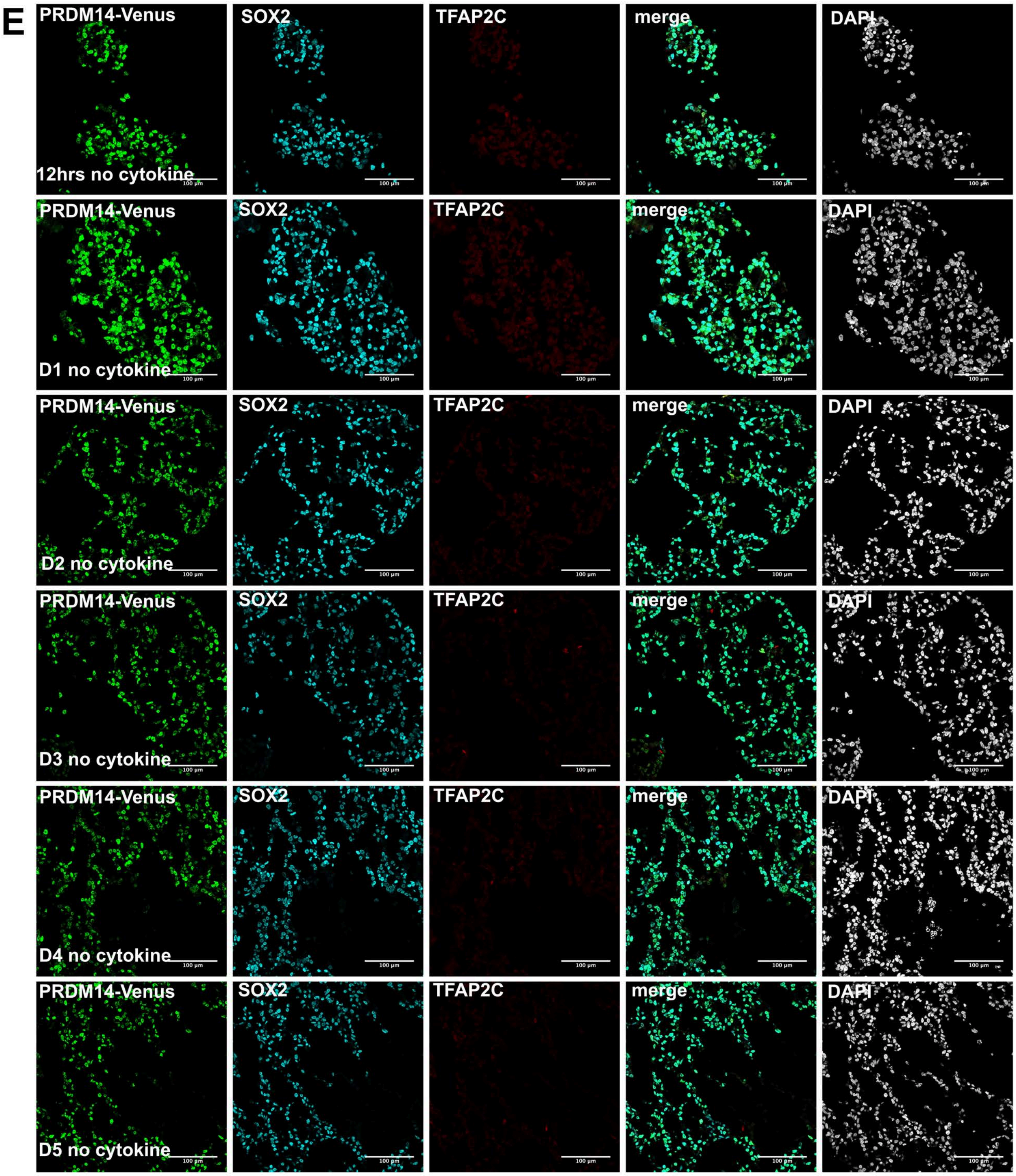

Supplementary Figure 2 (continued). Expression dynamics of PRDM14-Venus and other germ cell and pluripotency markers during hPGCLC specification. (E) Time-course IF showing TFAP2C, PRDM14-Venus and SOX2 expression in embryoid body (EB) sections throughout differentiation of PRDM14-AID-Venus cells without hPGCLC-inducing cytokines (representative of 1 experiment). Note that unlike normal induction with cytokines (Supplementary Figure 2F), “no cytokine” differentiation in the basal medium yields no hPGCLCs, but allows sustained SOX2 and PRDM14 expression. *(Supplementary Fig.2 is continued on the next page)*

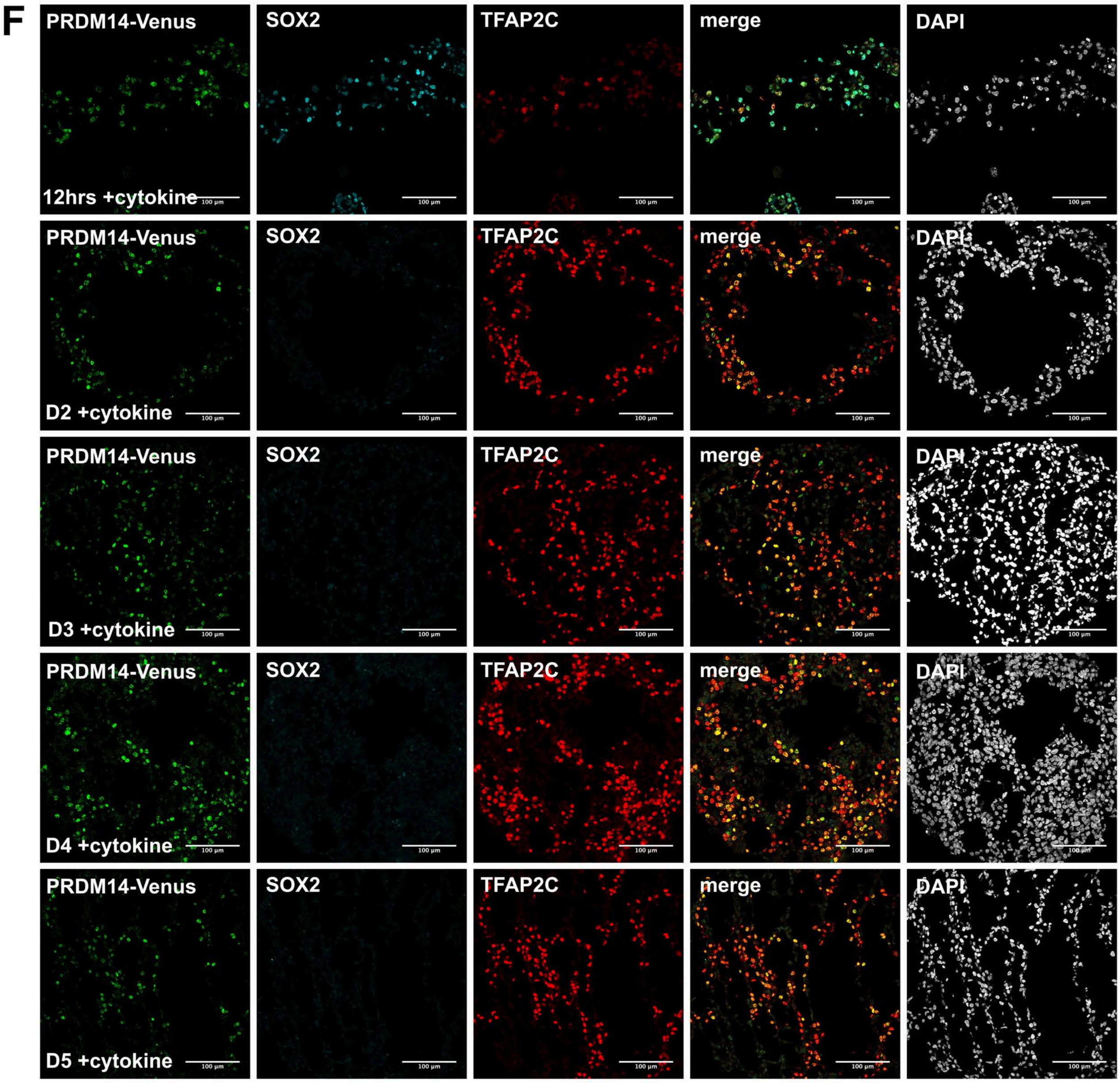

Supplementary Figure 2 (continued). Expression dynamics of PRDM14-Venus and other germ cell and pluripotency markers during hPGCLC specification. (F) Time-course IF showing TFAP2C, PRDM14-Venus and SOX2 expression in embryoid body (EB) sections throughout differentiation of PRDM14-AID-Venus cells with hPGCLC-inducing cytokines (representative of 1 experiment). *(Supplementary Fig.2 is continued on the next page)*

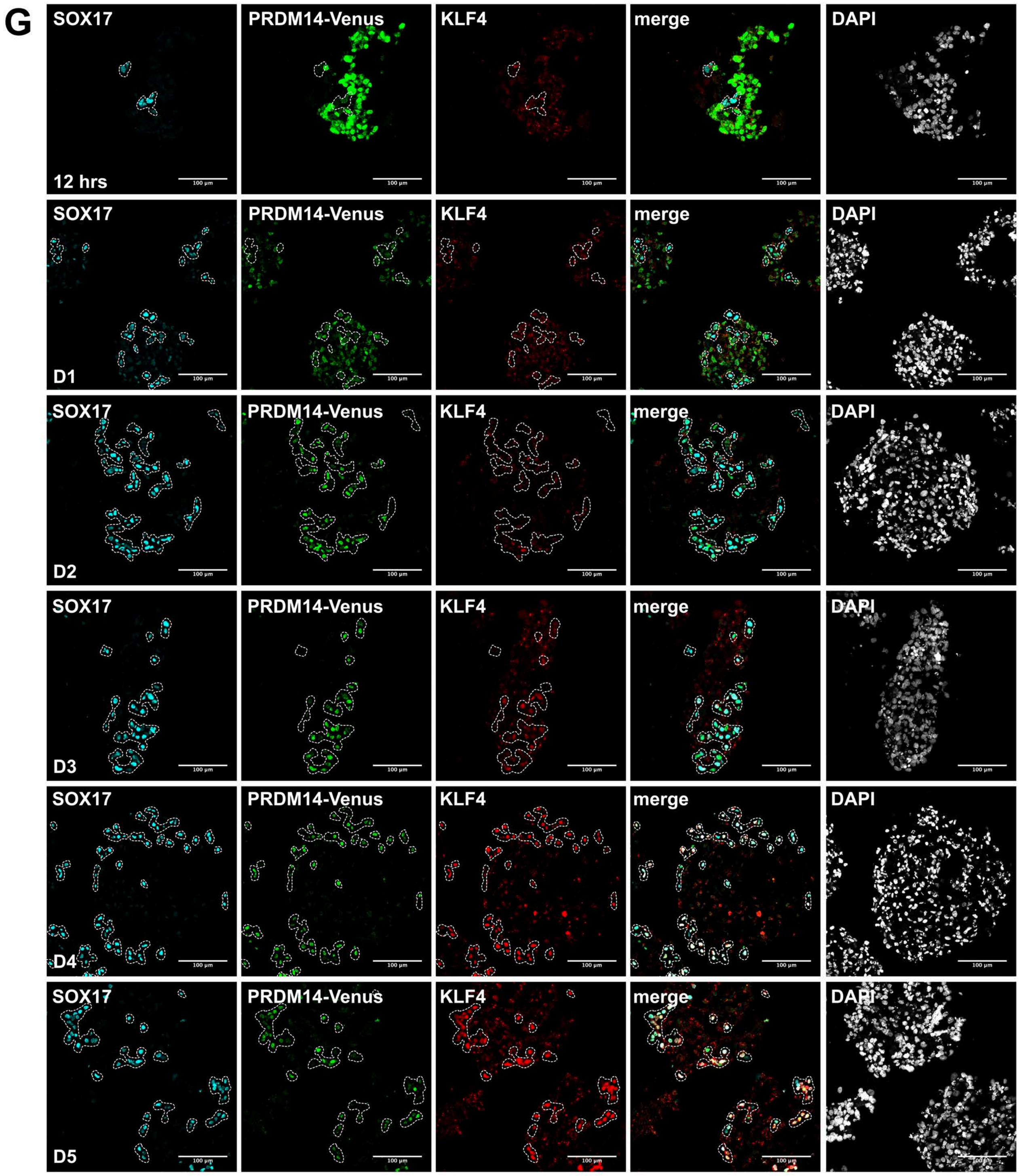

Supplementary Figure 2 (continued). Expression dynamics of PRDM14-Venus and other germ cell and pluripotency markers during hPGCLC specification. (G) Time-course IF showing SOX17, PRDM14-Venus and KLF4 expression (representative of 1 experiment). Dashed line highlights $SOX17^+$ cells. Note KLF4 upregulation from D3 of differentiation.

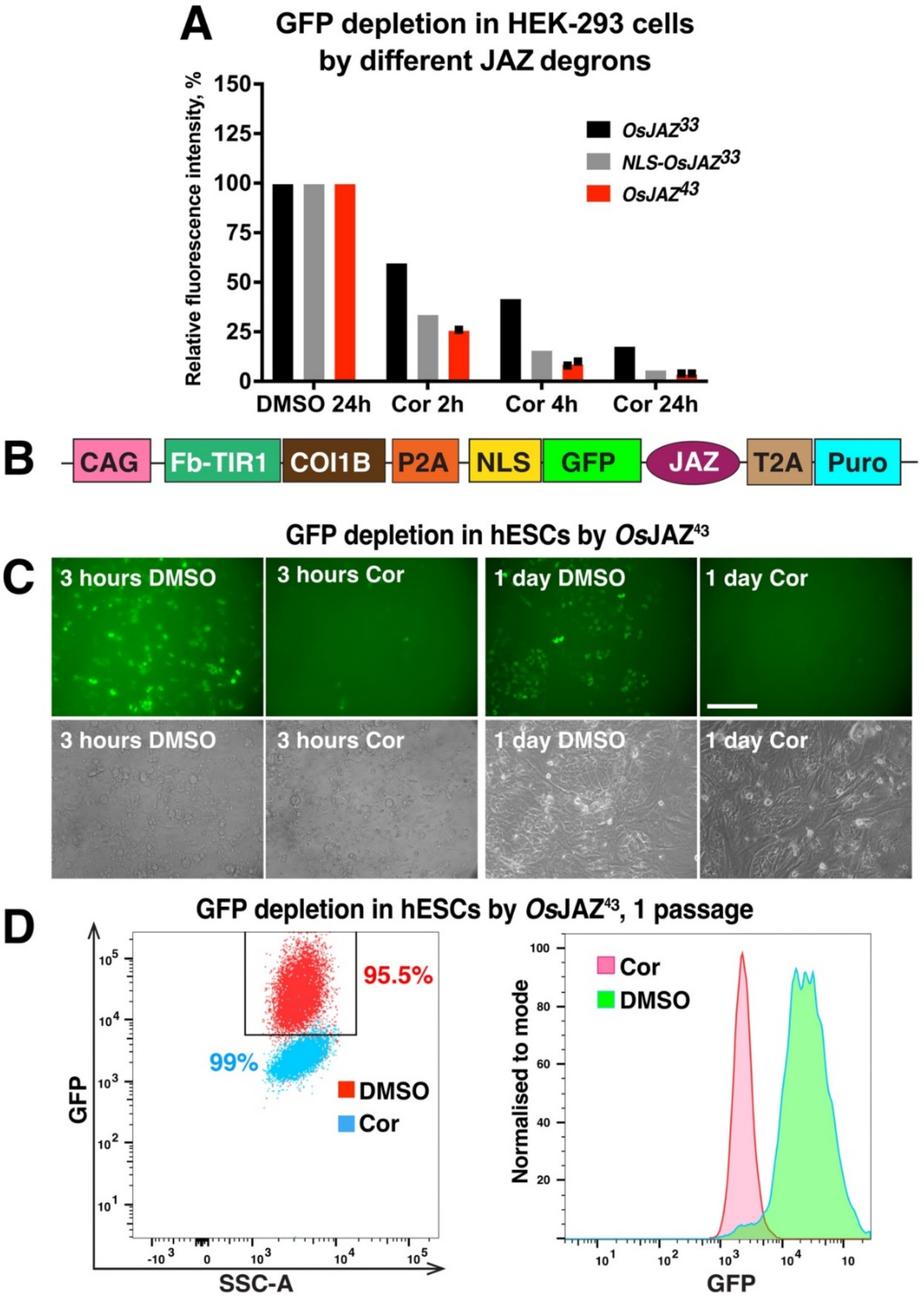

Supplementary Figure 3. Selection of JAZ degron sequence and its validation in hESCs. (A) GFP depletion in HEK-293T cells by three indicated JAZ degron versions. GFP fluorescence intensity was measured by flow cytometry after indicated times of Cor or DMSO (control) treatment. Data is from n=1 (for Cor 2h) or n=2 DMSO, Cor 4h and Cor 24h) independent experiments and is shown as relative geometric mean fluorescence intensity (normalised to DMSO control). **(B)** Scheme of the construct used to express GFP-JAZ in hESCs. COI1B (coronatine receptor) from rice (*Oryza sativa*) was fused to the F-box domain of TIR1 (Fb-TIR1). GFP fluorescent protein was fused to the JAZ degron. P2A and T2A – self-cleaving peptides. NLS – nuclear localisation signal. Puro – puromycin resistance gene. **(C)** Epifluorescence and bright-field photographs of GFP-JAZ hESCs after 3 hours or 1 day of Cor treatment (representative of 2 experiments). DMSO was used as a negative control for GFP depletion. Scale bar is 100 µm. **(D)** Flow cytometry plots confirming GFP-JAZ depletion after one passage in the presence of Cor.

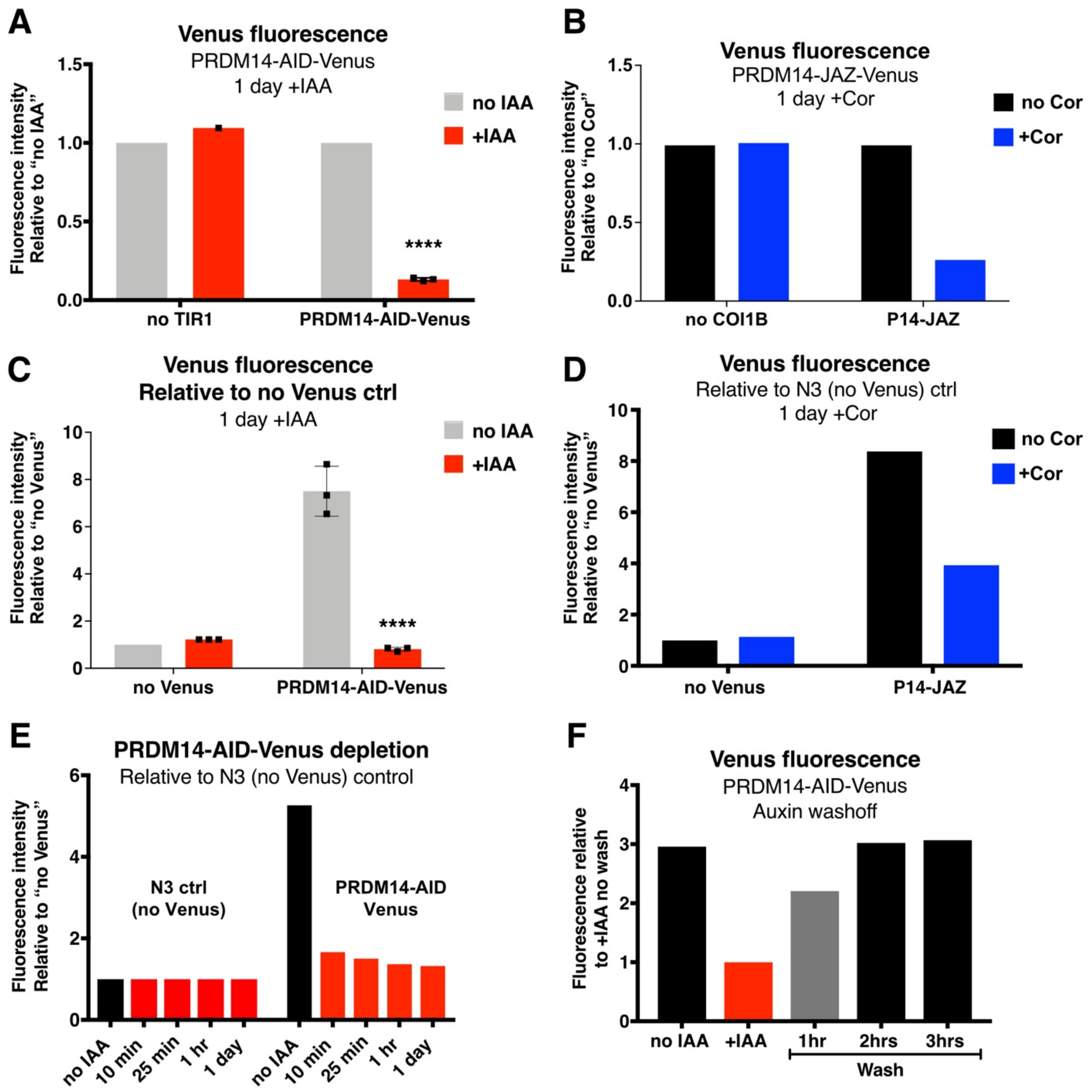

Supplementary Figure 4. Venus fluorescence quantification shows higher efficiency of AID compared to JAZ degron. (A) Related to Fig.3B. Mean fluorescence intensities (MFI) of PRDM14-AID-Venus cells and indicated controls grown with or without IAA for 1 day. To focus on pluripotent cells that would normally express PRDM14, fluorescence intensities were measured in the nuclei of NANOG-positive cells. A cell line lacking TIR1 hormone receptor ("no TIR1") was used as a negative control for PRDM14-AID-Venus depletion. Data show the mean n=3 (for PRDM14-AID-Venus) or n=1 (for "no TIR1") experiments +/- SD using >100 $NANOG^+$ cells per condition per replicate, **** P<0.0001 (two-way ANOVA followed by Sidak's multiple comparison test). **(B)** Related to Fig.3C. PRDM14-JAZ-Venus cells were grown with or without Cor for 1 days. To focus on pluripotent cells that would normally express PRDM14, fluorescence intensities were measured in the nuclei of OCT4-positive cells. A cell line lacking COI1B hormone receptor ("no COI1B") was used as a negative control for PRDM14-JAZ-Venus depletion. Data show mean of >100 $OCT4^+$ cells per condition from n=1 biological replicate. **(C)** Data from (A) normalised to a control cell line lacking AID-Venus KI. Data show the mean n=3 (for PRDM14-AID-Venus) or n=1 (for "no Venus") experiments +/- SD using >100 $NANOG^+$ cells per condition per replicate, **** P<0.0001 (two-way ANOVA followed by Sidak's multiple comparison test). **(D)** Data from (B) normalised to a control cell line lacking JAZ-Venus KI. Data show mean of >100 $OCT4^+$ cells per condition from n=1 biological replicate. **(E)** Kinetics of Venus fluorescence depletion in PRDM14-AID-Venus hESCs. hESCs were treated with IAA for indicated time periods. Homogeneous depletion with Venus intensity close to "no Venus control" was reached within 25 minutes, as indicated by IF staining in Fig.3B. Data show mean of >100 cells per condition from n=1 biological replicate. **(F)** Kinetics of Venus fluorescence restoration in PRDM14-AID-Venus hESCs. hESCs were treated with IAA for 2 days, washed and grown without IAA for indicated periods of time. Data show mean of >100 cells per condition from n=1 biological replicate.

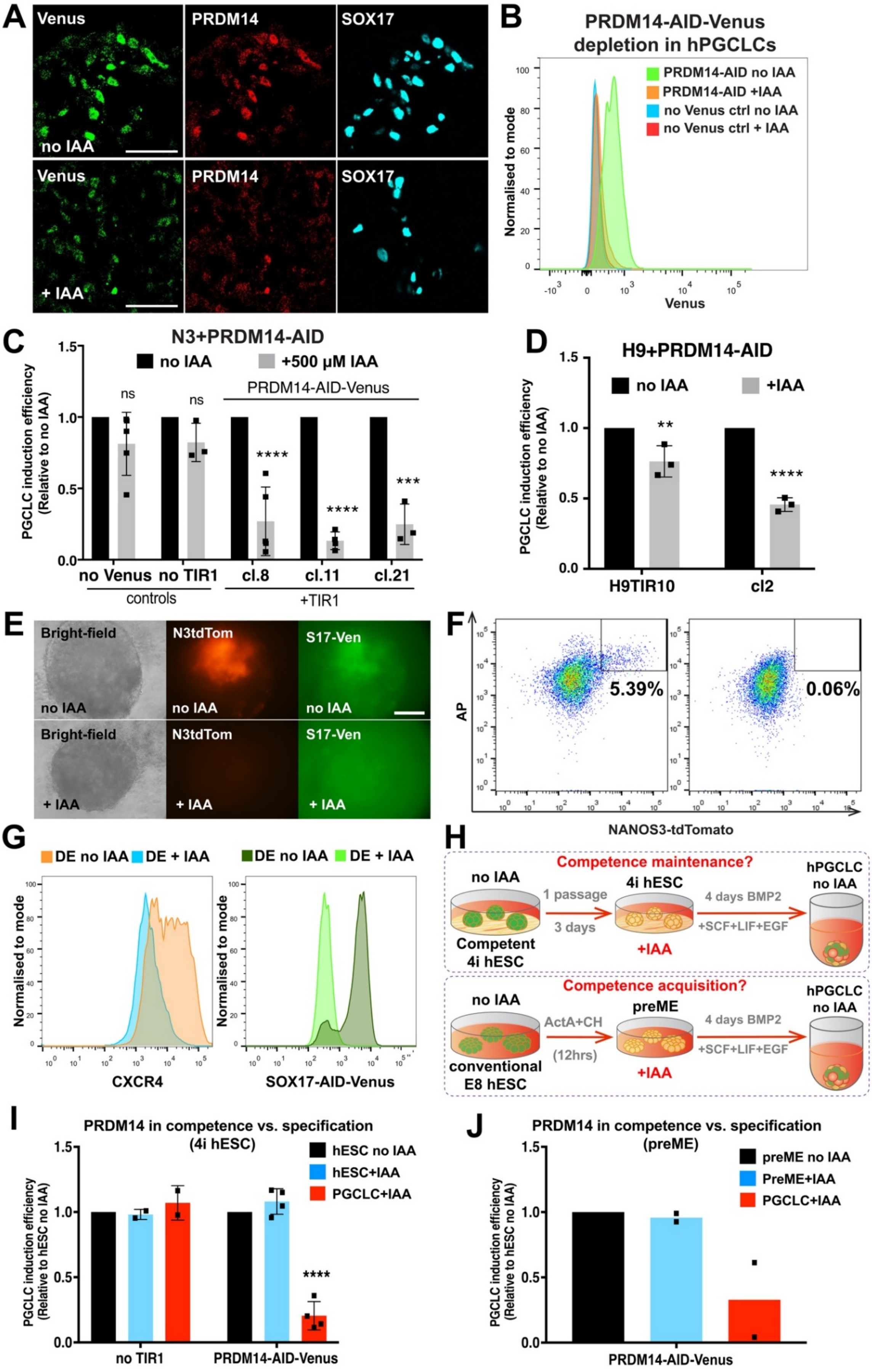

Supplementary Figure 5. AID system allows studying the roles of PRDM14 and SOX17 TFs in cell fate decisions. **(A)** IF analysis of PRDM14-AID-Venus depletion in D4 IAA-treated EBs (representative of 2 experiments). SOX17 marks hPGCLCs. Scale bar is 60 µm. **(B)** Representative flow plots showing Venus fluorescence in AP$^+$CD38$^+$ hPGCLCs induced with or without auxin. **(C)** hPGCLC induction efficiencies from PRDM14-AID-Venus differentiated with or without

500 μM IAA concentrations (note a different concentration compared to Fig.4B). The induction efficiency of untreated cells ("no IAA") was set to 1. Data show mean+/-SD for n=5 for N3, cl8 and cl11; n=3 for "no TIR1" and cl21; ns – not significant: P=0.1322 for "no Venus" and P=0.4129 for "no TIR1", **** P<0.0001 (Two-way ANOVA followed by Sidak's multiple comparison test). **(D)** PRDM14 depletion using AID in H9 hESC background also reduces hPGCLC specification. Note that this cell line lacks NANOS3-tdTomato reporter and hPGCLCs were identified by AP and CD38 staining. H9TIR10 is a parental cell line expressing TIR1 but lacking the AID-Venus KI. Data show mean+/-SD for n=3 independent experiments per clone, ** P=0.0028, **** P<0.0001 (Two-way ANOVA followed by Sidak's multiple comparison test). **(E)** Epifluorescence photographs show colocalization of NANOS3-tdTomato and SOX17-AID-Venus fluorescence in D4 EBs; both signals are reduced upon IAA treatment. Representative of 3 experiments. Scale bar is 100 μm. **(F)** Representative flow cytometry plots showing hPGCLC induction using the SOX17-AID-Venus cell line with or without IAA. **(G)** SOX17 degradation decreases definitive endoderm (DE) specification. Representative flow cytometry plots; DE cells are marked by CXCR4 expression. **(H)** Experimental workflow of PRDM14 depletion in competent hESCs, as well as during preME induction. PRDM14 role in competence maintenance was assessed by depleting it in competent (4i) hESCs, followed by washing off the IAA for PRDM14 restoration and inducing hPGCLCs (without IAA). To test if PRDM14 is required for competence acquisition, it was depleted during the generation of the preME competent cells. Auxin was subsequently removed and hPGCLCs were induced without IAA. **(I)** PRDM14-AID depletion in competent hESCs does not reduce hPGCLC induction therefrom. IAA was added for 1 passage (3 days) prior to hPGCLC induction and washed of before the onset of hPGCLC differentiation. hESCs grown without auxin were differentiated with or without IAA as above. Data show mean+/-SD for n=2 (no TIR1 control) and n=4 (PRDM14-AID) independent experiments, **** P<0.0001 (Two-way ANOVA followed by Sidak's multiple comparison test). **(J)** PRDM14-AID depletion during the acquisition of hPGC-competent state via pre-mesendoderm (preME) does not reduce hPGCLC induction therefrom. IAA was added for 12 hours during preME generation from conventional hESCs and washed off before the onset of hPGCLC differentiation. preME induced without auxin was differentiated with or without IAA as above. Data show mean from n=2 independent experiments.

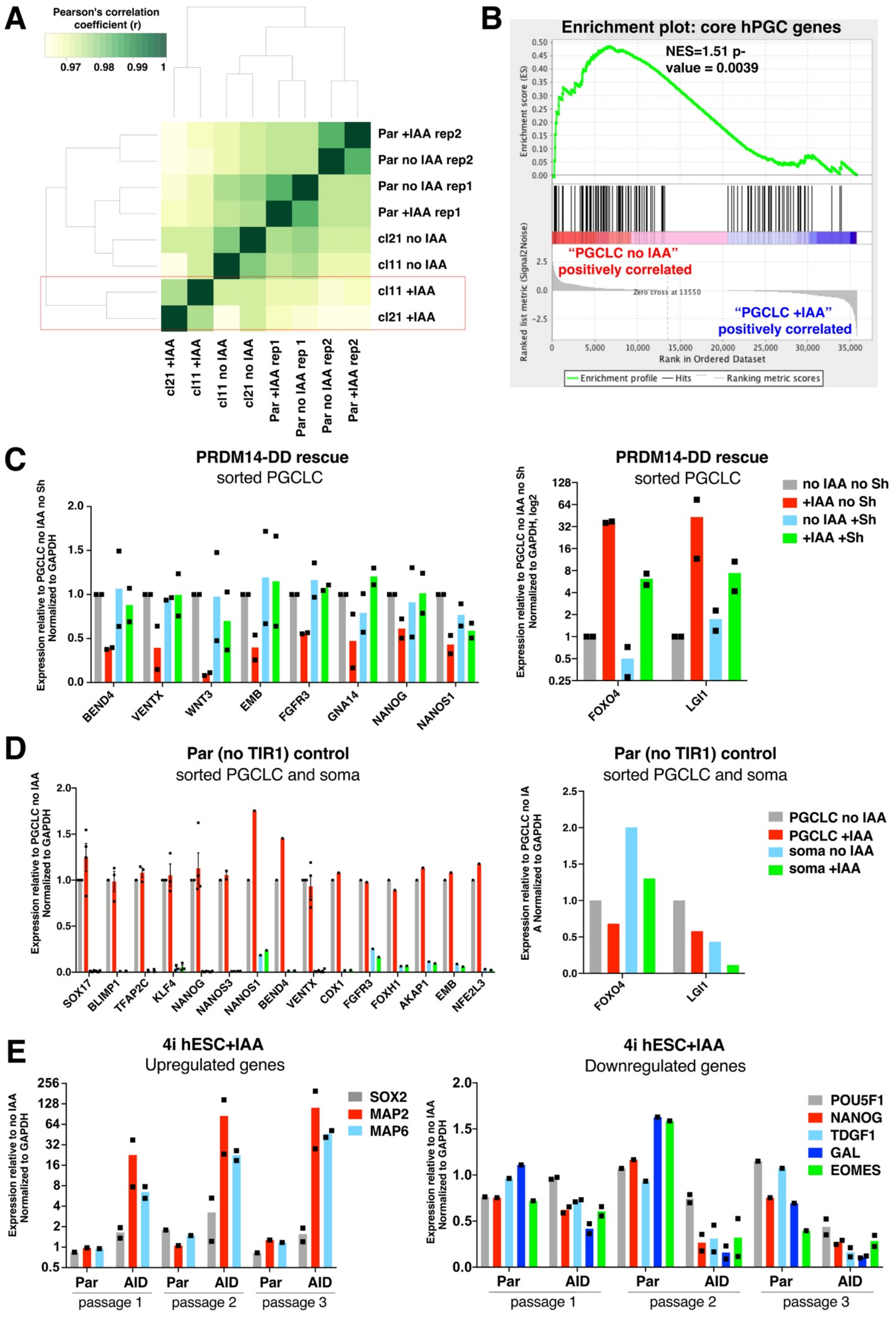

A
Pearson's correlation coefficient (r)
0.97 0.98 0.99 1
Par +IAA rep2
Par no IAA rep2
Par no IAA rep1
Par +IAA rep1
cl21 no IAA
cl11 no IAA
cl11 +IAA
cl21 +IAA
B
Enrichment plot: core hPGC genes
NES=1.51 p-value = 0.0039
Enrichment score (ES)
"PGCLC no IAA" positively correlated
"PGCLC +IAA" positively correlated
Zero cross at 13550
Ranked list metric (Signal2Noise)
Rank in Ordered Dataset
Enrichment profile — Hits — Ranking metric scores
C
PRDM14-DD rescue
sorted PGCLC
Expression relative to PGCLC no IAA no Sh Normalized to GAPDH
BEND4 VENTX WNT3 EMB FGFR3 GNA14 NANOG NANOS1
Expression relative to PGCLC no IAA no Sh Normalized to GAPDH, log2
FOXO4 LGI1
no IAA no Sh
+IAA no Sh
no IAA +Sh
+IAA +Sh
D
Par (no TIR1) control
sorted PGCLC and soma
Expression relative to PGCLC no IAA Normalized to GAPDH
SOX17 BLIMP1 TFAP2C KLF4 NANOG NANOS3 NANOS1 BEND4 VENTX CDX1 FGFR3 FOXH1 AKAP1 EMB NFE2L3
Expression relative to PGCLC no IA A Normalized to GAPDH
FOXO4 LGI1
PGCLC no IAA
PGCLC +IAA
soma no IAA
soma +IAA
E
4i hESC+IAA
Upregulated genes
Expression relative to no IAA Normalized to GAPDH
SOX2
MAP2
MAP6
Par AID Par AID Par AID
passage 1 passage 2 passage 3
4i hESC+IAA
Downregulated genes
POU5F1
NANOG
TDGF1
GAL
EOMES

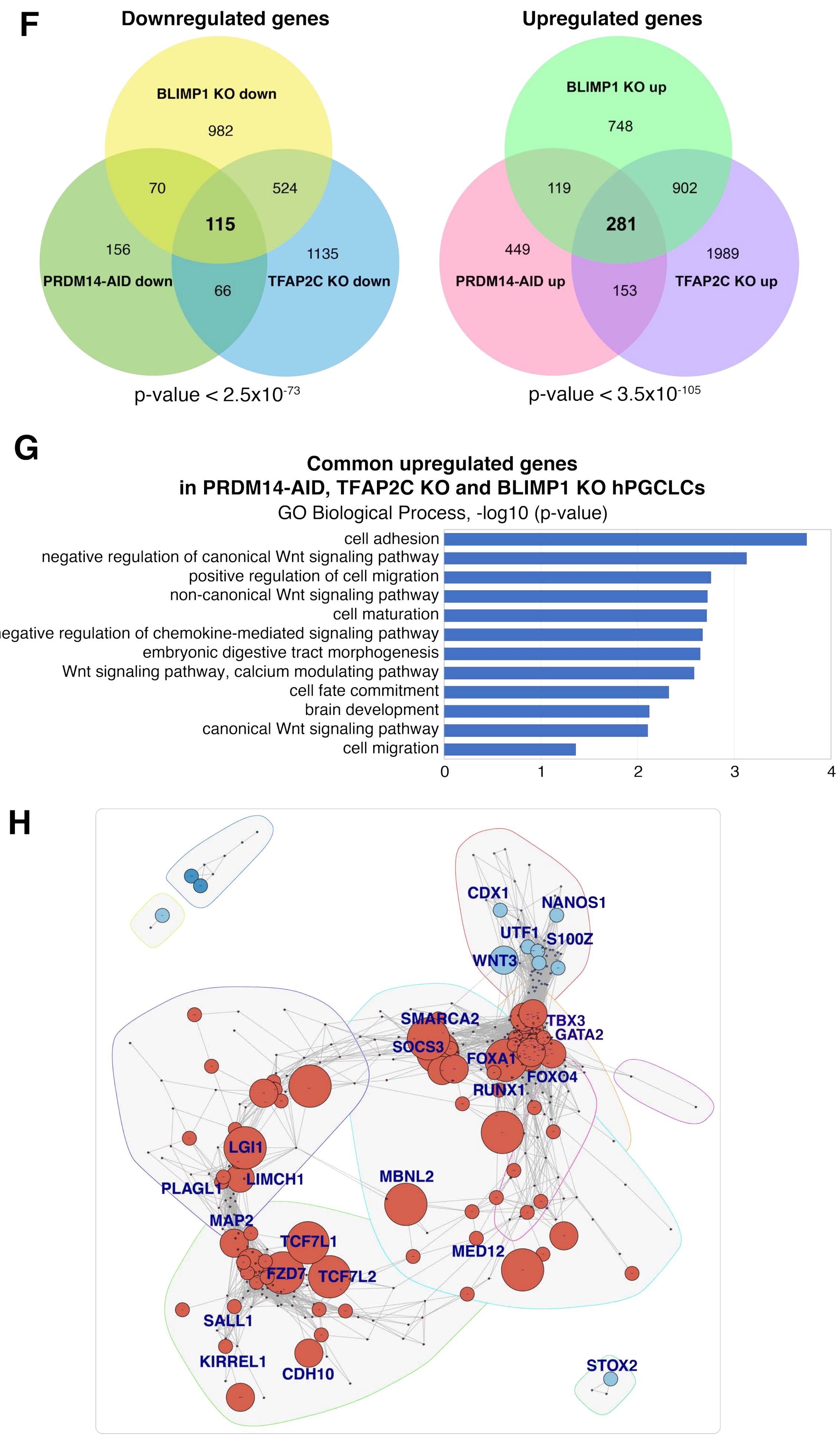
F
Downregulated genes
BLIMP1 KO down
982
70
524
115
156
1135
PRDM14-AID down
66
TFAP2C KO down
p-value < 2.5x10^-73
Upregulated genes
BLIMP1 KO up
748
119
902
281
449
1989
PRDM14-AID up
153
TFAP2C KO up
p-value < 3.5x10^-105
G
Common upregulated genes
in PRDM14-AID, TFAP2C KO and BLIMP1 KO hPGCLCs
GO Biological Process, -log10 (p-value)
cell adhesion
negative regulation of canonical Wnt signaling pathway
positive regulation of cell migration
non-canonical Wnt signaling pathway
cell maturation
negative regulation of chemokine-mediated signaling pathway
embryonic digestive tract morphogenesis
Wnt signaling pathway, calcium modulating pathway
cell fate commitment
brain development
canonical Wnt signaling pathway
cell migration
0
1
2
3
4
H
CDX1
NANOS1
UTF1
S100Z
WNT3
SMARCA2
TBX3
GATA2
SOCS3
FOXA1
FOXO4
RUNX1
LGI1
PLAGL1
LIMCH1
MBNL2
MAP2
TCF7L1
FZD7
TCF7L2
MED12
SALL1
KIRREL1
CDH10
STOX2

Supplementary Figure 6. Transcriptional changes in hPGCLCs induced without PRDM14. (A) Heatmap showing Pearson's correlation between hPGCLC RNA-seq samples. "Par" denotes parental control lacking TIR1 and hence unresponsive to auxin; "r" – Pearson's correlation coefficient. PRDM14-depleting clones cluster separately and are highlighted in red. **(B)** Gene set enrichment analysis (GSEA) of 123 hPGC-specific genes on the transcriptome of hPGCLCs specified with or without IAA. PRDM14-deficient hPGCLCs ("PGCLC+IAA") are negatively correlated with the gene set, if compared against "PGCLC no IAA" control. **(C)** qPCR shows transcriptional rescue of tested DEGs by ectopic PRDM14-DD. hPGCLCs were differentiated with or without IAA and/or Shield-1 (Sh) and sorted as NANOS3-tdTomato^{+}AP^{+} cells; soma denotes the double-negative population from the same experiments. Data show gene expression relative to no IAA no Sh PGCLCs and normalised to *GAPDH*, mean of n=2 independent experiments. **(D)** Parental ("no TIR1") control for qPCR validation of RNA-seq (see Fig.6G). hPGCLCs were sorted as NANOS3-tdTomato^{+}AP^{+} cells, while soma denotes the double-negative population from the same experiments. Data show gene expression relative to no IAA hPGCLCs and normalised to *GAPDH*, mean+/-SEM of n=4 (*SOX17, NANOG, VENTX*) mean +/-SEM of n=3 (*BLIMP1, TFAP2C, KLF4*), mean of n=2 (*NANOS3*) or from of n=1 (other genes) independent experiments, all not significant by multiple unpaired t-tests (two-tailed P>0.18). **(E)** qPCR on unsorted PGC-competent (4i) PRDM14-AID hESCs cultured with or without IAA for 3 consecutive passages. The parental cell line lacking TIR1 is shown as a control. Data show mean of n=2 hormone-sensitive clones; n=1 for the parental control. Note downregulation of pluripotency genes and upregulation of pro-neuronal differentiation transcripts. **(F)** Venn diagrams highlighting significant overlap between transcriptional changes in hPGCLCs lacking PRDM14, TFAP2C or BLIMP1 (relative to corresponding WT controls). P-values were obtained using a modified hypergeometric test (Polanski et al., 2014). **(G)** GO analysis performed on shared upregulated genes. The graph shows top non-redundant GO terms as –log10(p-value). **(H)** Co-expression network of differentially expressed genes in hPGCLCs (+IAA vs no IAA, log2FC > 1.5, p-value < 0.05). Nodes were scaled according to the number of PRDM14 ChIP-seq peaks assigned to the gene. The network was partitioned into overlapping communities using the Louvain method (see Methods). Representative direct targets of PRDM14 were labelled. Upregulated genes were highlighted in orange and downreguated in blue. Note that downregulated PRDM14 targets form a separate co-expression cluster.

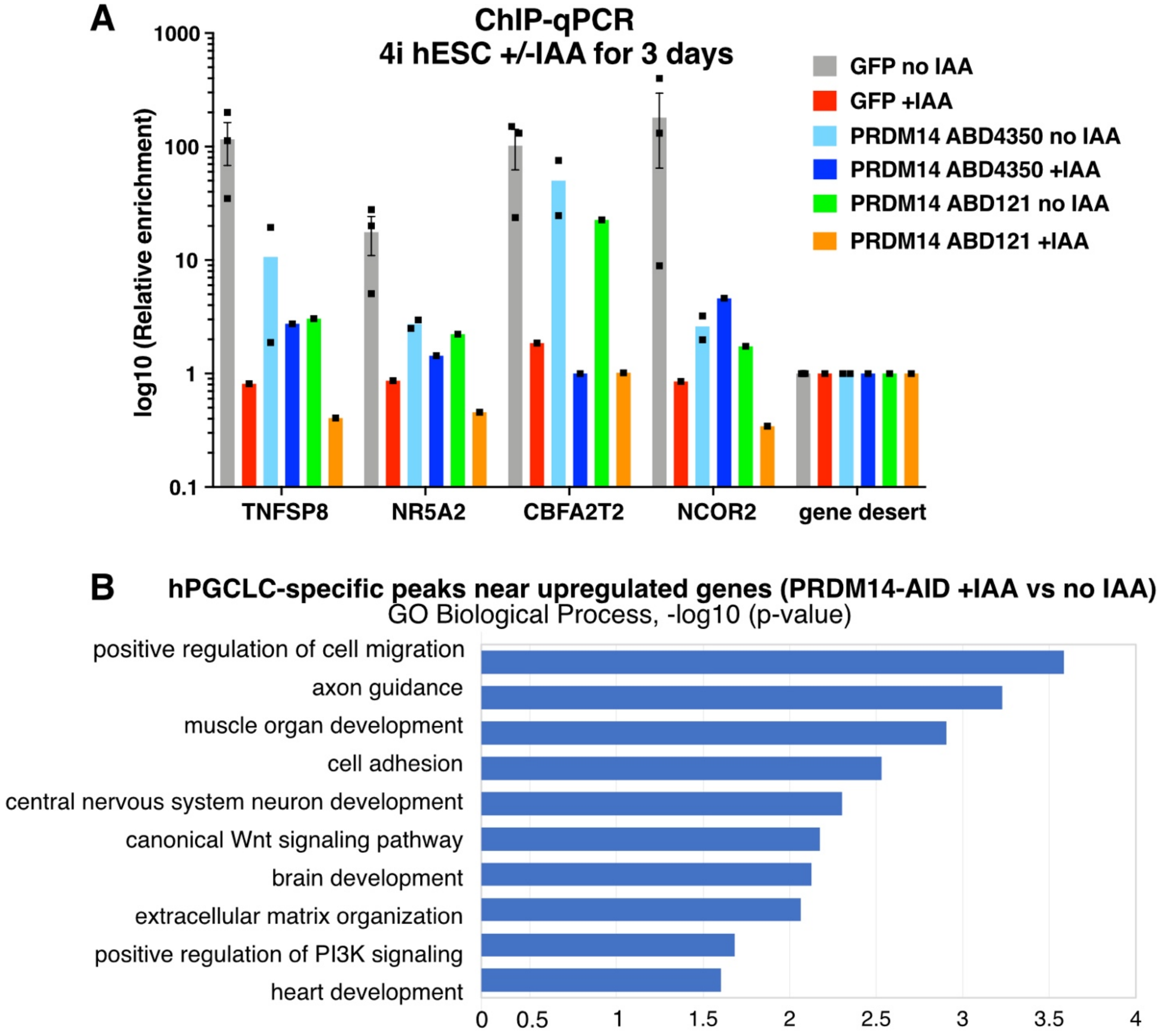

Supplementary Figure 7. PRDM14-Venus ChIP optimisation and GO analysis on direct targets of PRDM14. (A) ChIP-qPCR in competent PRDM14-AID-Venus hESCs using different antibodies. Anti-GFP antibody allows higher enrichment than the tested anti-PRDM14 antibodies. The primers were designed to span selected PRDM14 ChIP-seq peaks in conventional hESCs from (Chia et al. 2010). Data show log10 enrichment normalised to input and relative to a gene desert region (control), mean+/-SEM for n=1 (GFP+IAA, PRDM14 ABD4350 +IAA, PRDM14 ABD121 no IAA, PRDM14 ABD121 +IAA), n=2 (PRDM14 ABD4350 no IAA), n=3 (GFP no IAA) independent experiments. **(B)** GO analysis on hPGCLC-specific PRDM14 peaks (q-value<0.05) near genes upregulated in hPGCLCs upon PRDM14 depletion. Top non-redundant GO terms are shown as –log10(p-value).

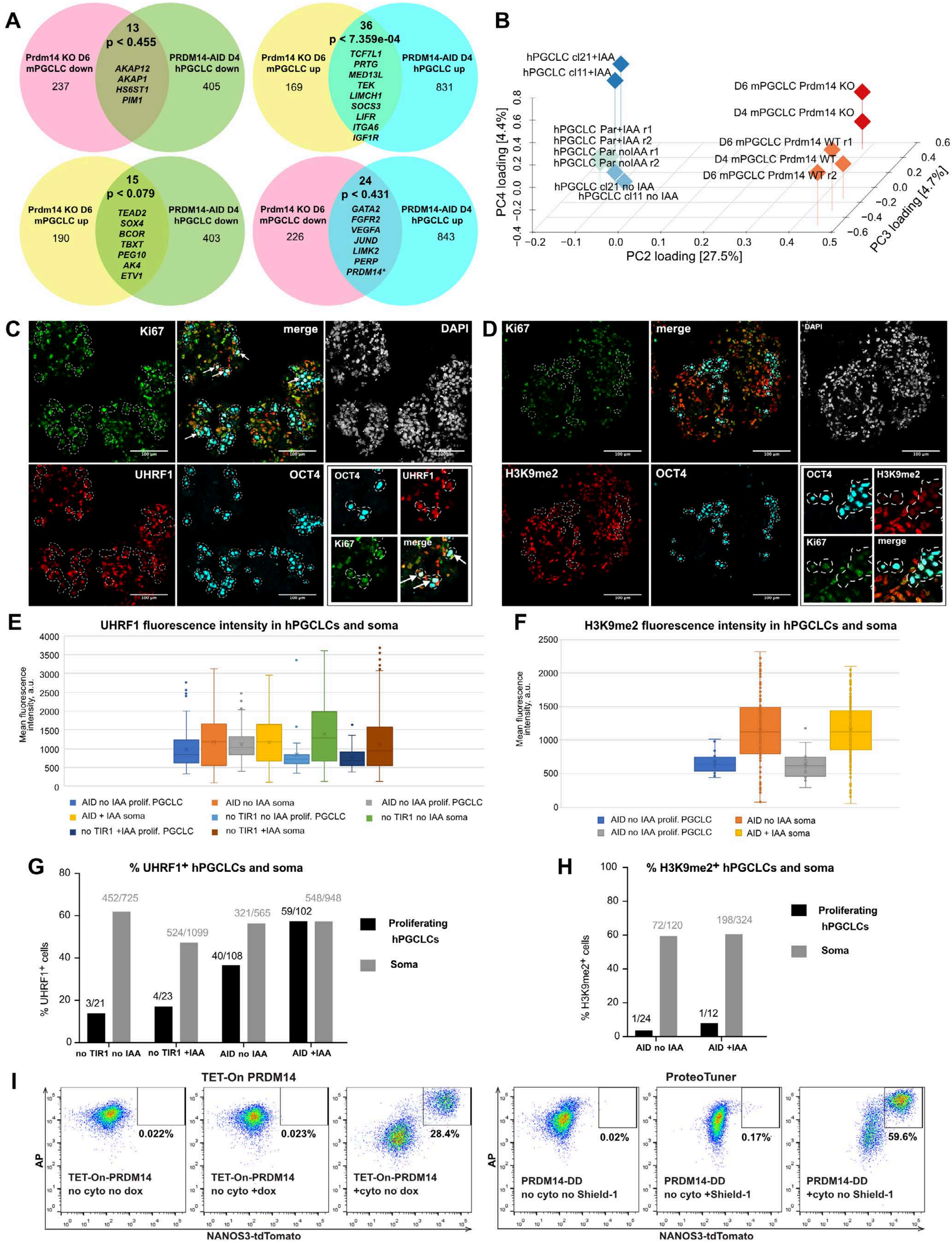

Supplementary Figure 8. Comparison of molecular functions of PRDM14 in mouse and human PGCLCs. (A) Venn diagrams comparing transcriptional changes in *Prdm14*$^{-/-}$ mouse PGCLCs and PRDM14-AID hPGCLCs (+IAA) relative to corresponding WT controls. P-values were obtained by pairwise hypergeometric tests. Examples of overlapping DEGs are shown. * In PRDM14-AID hPGCLCs PRDM14 is depleted only at the protein level, while the transcript is still present and even upregulated, presumably due to autoregulation. **(B)** 3D PCA plot showing distinct transcriptional response of mouse and human PGCLCs to the absence of PRDM14. Data from Supplementary Data 4 with logFC>1 were used. Note that mouse WT and KO samples separate by PC4, while human samples induced with or without PRDM14 separate along

PC3. Par – parental control lacking TIR1; r1 and r2 – replicates 1 and 2. **(C)** IF analysis of UHRF1 expression in D4 PRDM14-AID EBs induced in the presence of auxin (representative of 2 experiments). hPGCLCs are marked by OCT4, proliferating cells are marked by Ki-67. OCT4-positive cells are highlighted by a dashed line. White arrows show examples of PRDM14-deficient proliferating hPGCLC that retain UHRF1. Scale bar is 100 µm. **(D)** IF analysis of H3K9me2 levels in D4 PRDM14-AID EBs (representative of 1 experiment). hPGCLCs are marked by OCT4, proliferating cells are marked by Ki-67. OCT4-positive cells are highlighted by a dashed line. Scale bar is 100 µm. **(E)** UHRF1 MFI in D4 EBs. Proliferating hPGCLCs were identified as $OCT4^{+}Ki67^{+}$ cells. Note that UHRF1 fluorescence intensities in hPGCLCs+IAA and soma+IAA were normalised by the ratio of median fluorescence in "soma+IAA" to "soma no IAA". The box and whisker plots show MFI of n>20 cells per condition (see (G)) from n=1 staining. The cross (x) in the box shows the mean, while the line shows the median of the dataset. The distance from the bottom to the upper line of the box shows the interquartile range (IQR). Quartiles were calculated using the exclusive median method. The whiskers show +/- 1.5×IQR with any data points below or above the whiskers considered as outliers. **(F)** H3K9me2 MFI in D4 EBs. Proliferating hPGCLCs were identified as $OCT4^{+}Ki67^{+}$ cells. Note that H3K9me2 fluorescence intensities in hPGCLCs+IAA and soma+IAA were normalised by the ratio of median fluorescence in "soma+IAA" to "soma no IAA". The box and whisker plots show MFI of n>10 cells per condition (see (H)) from n=1 staining. The cross (x) in the box shows the mean, while the line shows the median of the dataset. The distance from the bottom to the upper line of the box shows the interquartile range (IQR). Quartiles were calculated using the exclusive median method. The whiskers show +/- 1.5×IQR with any data points below or above the whiskers considered as outliers. **(G)** Quantification of UHRF1-positive cells in proliferating hPGCLCs and soma of PRDM14-AID and control EBs. Normalised fluorescence values were used. Numbers of UHRF1 positive and total cells counted are shown for each sample. **(H)** Quantification of H3K9me2-positive cells in proliferating hPGCLCs and soma of PRDM14-AID EBs. Normalised fluorescence values were used. Numbers of H3K9me2 positive and total cells counted are shown for each sample. **(I)** Representative (of 3 experiments) flow cytometry plots showing that ectopic PRDM14 induced by either dox or Shield-1 does not induce hPGCLC induction without BMP2 and other cytokines ("no cyto"). Dox or Shield-1 were added on D0 of differentiation.

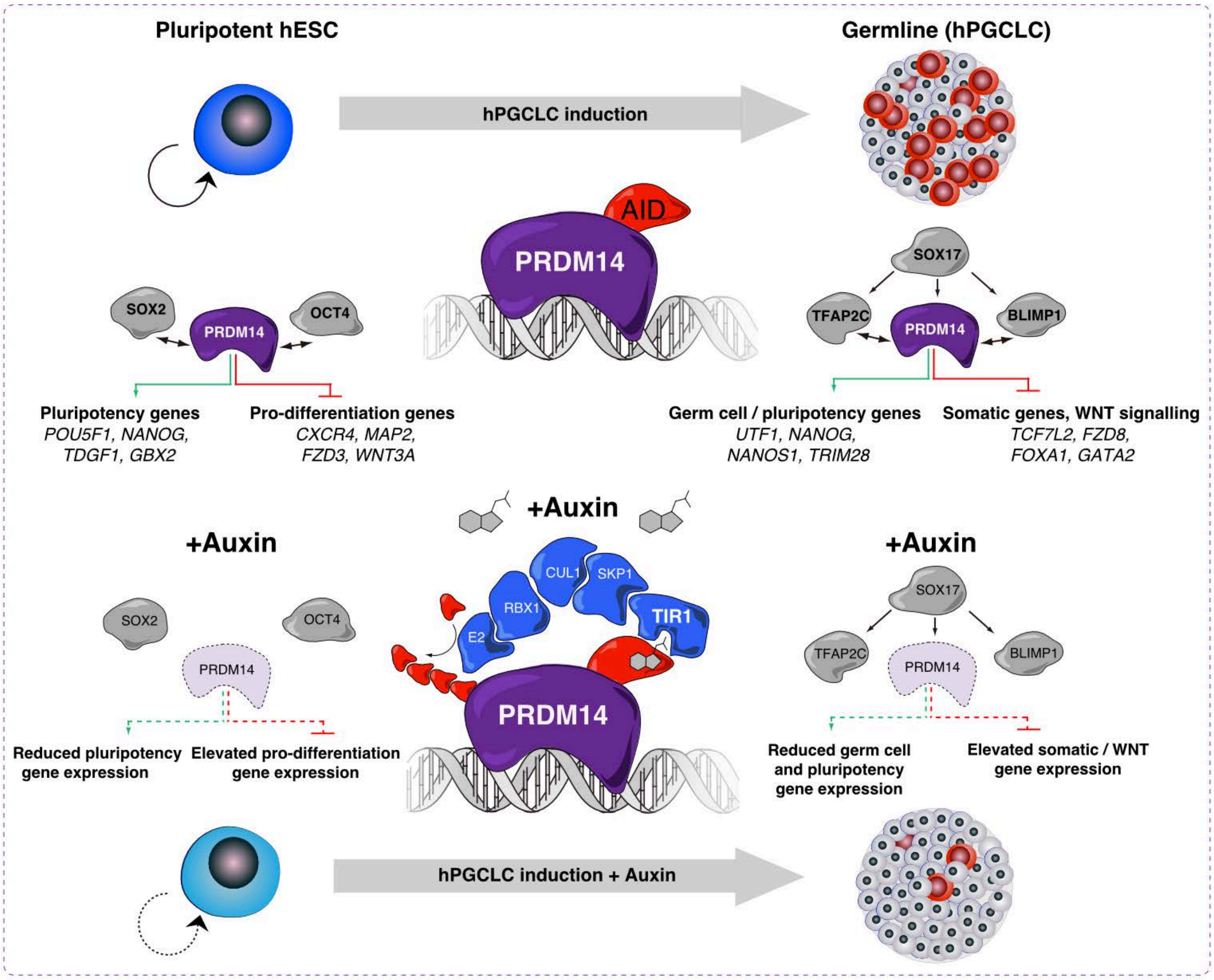

Supplementary Figure 9. Graphical summary of key findings. Model summarising the context-dependent PRDM14 roles in human pluripotency and hPGCLC specification. Using PRDM14-AID-Venus we were able to map PRDM14 binding in hESCs and hPGCLCs, as well as deplete the protein with high time resolution. This showed that PRDM14 regulates target genes through promoter binding together with SOX2/OCT4 in hESCs or TFAP2C and BLIMP1 in hPGCLCs. PRDM14, TFAP2C and BLIMP1 are all downstream of SOX17. In hESCs, PRDM14 activates the expression of pluripotency genes and represses pro-differentiation genes. Similarly, in hPGCLCs, PRDM14 represses somatic transcripts, but activates germ cell- and pluripotency-related genes. Representative examples of activated and repressed genes are shown. When auxin is added PRDM14-AID fusion protein is recruited to the E3 ubiquitin ligase via an F-box auxin receptor TIR1. It leads to rapid protein ubiquitination and degradation, which allows time-resolved functional studies.

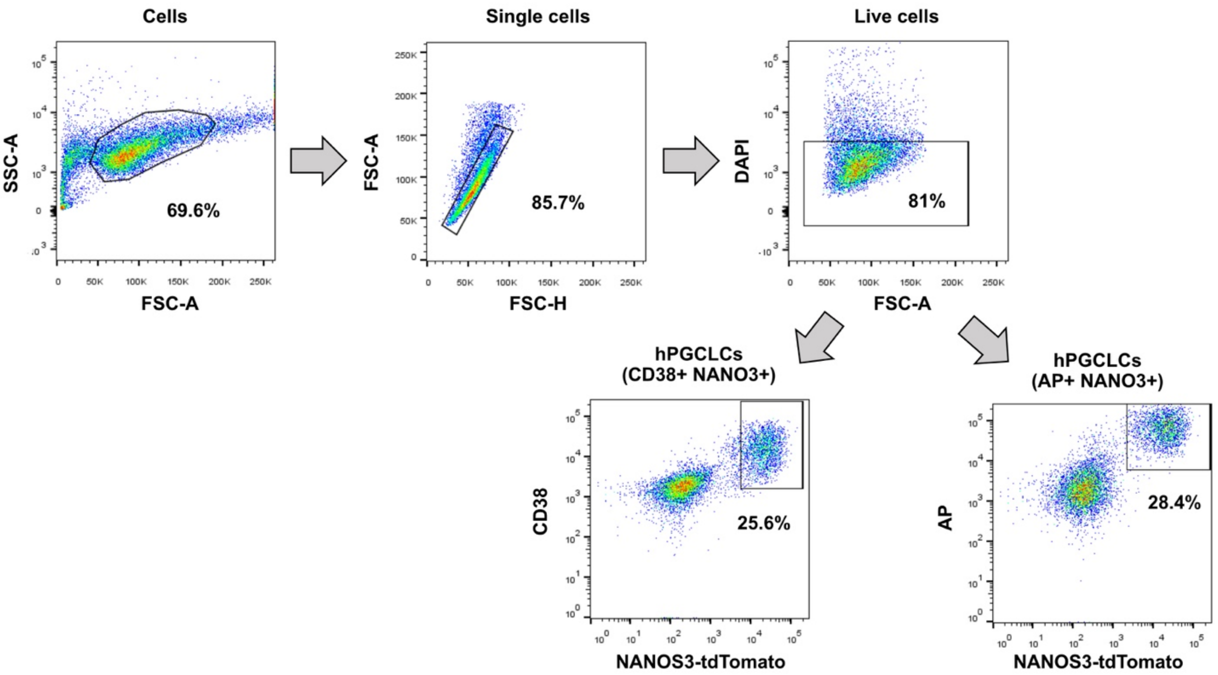

Supplementary Figure 10. Flow cytometry and FACS gating strategy to identify hPGCLCs. The gating was performed as follows: the initial cell population was defined as the largest compact events cluster on the SSC-A vs FSC-A plot; then cell doublets were eliminated using FSC-A vs FSC-H plot; afterwards dead cells were eliminated based on DAPI-positivity; finally, gating on live (DAPI-negative) cells, hPGCLCs were defined as NANOS3-tdTomato^{+}AP^{+} (or NANOS3-tdTomato^{+}CD38^{+}) cells. The boundaries between positive and negative gates were defined using unstained samples and negative controls (such as differentiation without cytokines).

Supplementary Tables

Supplementary Table 1. Mesendoderm (ME) medium composition.

Component	Final Concentration	Supplier
Advanced RPMI 1640	-	GIBCO
B27 supplement	1%	GIBCO
L-glutamine	2 mM	GIBCO
Nonessential amino acids	0.1 mM	GIBCO
Penicillin-Streptomycin	100 U/ml (Penicillin)<br>0.1 mg/ml (Streptomycin)	GIBCO
Activin A	100 ng/ml	SCI
CHIR99021	3 µM	Miltenyi Biotec
ROCK inhibitor (Y-27632)	10 µM	TOCRIS bioscience

Supplementary Table 2. Definitive endoderm (DE) medium composition.

Component	Final Concentration	Supplier
Advanced RPMI 1640	-	GIBCO
B27 supplement	1%	GIBCO
L-glutamine	2 mM	GIBCO
Nonessential amino acids	0.1 mM	GIBCO
Penicillin-Streptomycin	100 U/ml (Penicillin)<br>0.1 mg/ml (Streptomycin)	GIBCO
Activin A	100 ng/ml	SCI
BMP inhibitor (LDN193189)	0.5 µM	Sigma
ROCK inhibitor (Y-27632)	10 µM	TOCRIS bioscience

Supplementary Table 3. Antibodies used in the study.

Name	Used for	Dilution	Manufacturer	Validation
Alkaline Phosphatase PerCP-Cy5.5 #561508	Flow cytometry	0.5 µl/6 EBs	BD Pharmingen	Irie N *et al.* 2015[1]: FACS purification of human PGCLCs
Alkaline Phosphatase AF647 #561500	Flow cytometry	0.5 µl/6 EBs	BD Pharmingen	Irie N *et al.* 2015[1]: FACS purification of human PGCLCs
CD38 PerCP-Cy5.5 #303522	Flow cytometry	0.5 µl/6 EBs	BioLegend	Irie N *et al.* 2015[1]: FACS purification of human PGCLCs
CD38 AF647 #303514	Flow cytometry	0.5 µl/6 EBs	BioLegend	Irie N *et al.* 2015[1]: FACS purification of human PGCLCs
Alkaline Phosphatase AF488 #561495	Flow cytometry	5 µl/genital ridge	BD Pharmingen	Tang WW *et al.* 2015[2]: FACS purification of human PGCs
cKIT (CD117) PerCP-Cy5.5 #333950	Flow cytometry	25 µl/genital ridge	BD Pharmingen	Tang WW *et al.* 2015[2]: FACS purification of human PGCs
PRDM14 AB4350	ChIP and IF	1:100 for IF; 3 µl / 2.5 million cells in ChIP	Millipore	IF: Irie N *et al.* 2015[1]: IF on human PGCLCs; ChIP: antibody was validated in this manuscript by ChIP qPCR on PRDM14-target and control regions (Chia *et al.* 2010[3]) in wild type and PRDM14-depleted hESCs (Supplementary Fig.7a).
OCT4 #611203	IF	1:200	BD Biosciences	Tang WW *et al.* 2015[2]: IF on human PGCs
SOX17 AF1924	IF	1:500	R&D Systems	Tang WW *et al.* 2015[2]: IF on human PGCs
GFP ab13970	IF	1:1000	Abcam	Manufacturer validated by IF on GFP-transfected NIH/3T3 cells
PRDM14 ABD121	ChIP	3 µl / 2.5 million cells	Millipore	Antibody was validated in this manuscript by ChIP qPCR on PRDM14-target and control regions (Chia *et al.* 2010[3]) in wild type and PRDM14 depleted hESCs (Supplementary Fig.7A).
GFP ab290	ChIP	0.5 µl / 2.5 million cells	Abcam	Antibody was validated in this manuscript by ChIP qPCR on PRDM14-target and control regions (Chia *et al.* 2010[3]) in wild type and PRDM14 depleted hESCs (Supplementary Fig.7A).
TFAP2C sc-8977	IF	1:100	Santa Cruz	Tang WW *et al.* 2015[2]: IF on human PGCs
BLIMP1 #14-5963	IF	1:50	eBioscience	Tang WW *et al.* 2015[2]: IF on human PGCs
SOX2 sc-17320	IF	1:200	Santa Cruz	Irie N *et al.* 2015[1]: IF on human PGCLCs
myc-tag #2276	IF	1:4000	CST	Manufacturer validated by IF on COS cells transfected with a myc-tagged protein
HA-tag #3724	IF	1:200	CST	Manufacturer validated by IF on COS cells, transfected with an HA-tagged protein
V5-tag ab27671	IF	1:100	Abcam	Manufacturer validated by IF on CHO cells overexpressing a V5-tagged protein
Ki-67 ab16667	IF	1:500	Abcam	Irie N *et al.* 2015[1]: IF on human PGCLCs
NANOG ab80892	IF	1:100	Abcam	Manufacturer validated by IF on mES cells
UHRF1 #61342	IF	1:500	Active Motif	Tang WW *et al.* 2015[2]: IF on human PGCs
Alexa fluorophore (AF-488, 568 and/or 647)-conjugated secondary antibodies	IF	1:500	Invitrogen (ThermoFisher)	Irie N *et al.* 2015[1]: IF on human ESCs, PGCs and PGCLCs

Supplementary References

1. Irie, N. *et al.* SOX17 is a critical specifier of human primordial germ cell fate. *Cell* **160**, 253-268 (2015).
2. Tang, W.W. *et al.* A Unique Gene Regulatory Network Resets the Human Germline Epigenome for Development. *Cell* **161**, 1453-1467 (2015).
3. Chia, N.Y. *et al.* A genome-wide RNAi screen reveals determinants of human embryonic stem cell identity. *Nature* **468**, 316-320 (2010).
