# Supplementary material for: A critical role of PRDM14 in human primordial germ cell fate revealed by inducible degrons
Source: Nat Commun. 2020 Mar 9;11:1282. doi: 10.1038/s41467-020-15042-0 (PMC7062732; doi:10.1038/s41467-020-15042-0)
Supplement: Supplementary file 3 — Reporting Summary [file 41467_2020_15042_MOESM3_ESM.pdf]

nature research

Corresponding author(s): Azim M. Surani

Last updated by author(s): Dec 13, 2019

Reporting Summary

Nature Research wishes to improve the reproducibility of the work that we publish. This form provides structure for consistency and transparency in reporting. For further information on Nature Research policies, see Authors & Referees and the Editorial Policy Checklist .

Statistics

For all statistical analyses, confirm that the following items are present in the figure legend, table legend, main text, or Methods section.

n/a	Confirmed	
☐	☒	The exact sample size (n) for each experimental group/condition, given as a discrete number and unit of measurement
☐	☒	A statement on whether measurements were taken from distinct samples or whether the same sample was measured repeatedly
☐	☒	The statistical test(s) used AND whether they are one- or two-sided<br>*Only common tests should be described solely by name; describe more complex techniques in the Methods section.*
☐	☒	A description of all covariates tested
☐	☒	A description of any assumptions or corrections, such as tests of normality and adjustment for multiple comparisons
☐	☒	A full description of the statistical parameters including central tendency (e.g. means) or other basic estimates (e.g. regression coefficient) AND variation (e.g. standard deviation) or associated estimates of uncertainty (e.g. confidence intervals)
☐	☒	For null hypothesis testing, the test statistic (e.g. F, t, r) with confidence intervals, effect sizes, degrees of freedom and P value noted<br>*Give P values as exact values whenever suitable.*
☒	☐	For Bayesian analysis, information on the choice of priors and Markov chain Monte Carlo settings
☒	☐	For hierarchical and complex designs, identification of the appropriate level for tests and full reporting of outcomes
☐	☒	Estimates of effect sizes (e.g. Cohen's d, Pearson's r), indicating how they were calculated

Our web collection on statistics for biologists contains articles on many of the points above.

Software and code

Policy information about availability of computer code

Data collection

No software was used for data collection

Data analysis

All software used for data analysis is specified in the relevant sections of Materials and Methods.

- Flow cytometry data were analysed using FlowJo (version 10.0.7) from Tree Star.

- Confocal images were analysed using Fiji (version 2.0.0). A custom script for Fiji was used to segment nuclei with an area of 30-300 μm² in the DAPI channel and measure fluorescence intensity in other channels. The script is available at https://github.com/gurdon-institute/SOX17_PRDM14_Measurement.

- Multiple types of data (qPCR, fluorescent intensities, transcriptional and ChIP-seq) were also processed in Microsoft Excel (version 16.29).

- Statistical analyses were performed using Microsoft Excel (version 16.29), R (version 3.2.3) and GraphPad Prism 7 (version 7.0d).

- Graphs were plotted using ggplot2 (version 3.2.1) for R and GraphPad Prism 7.

- DNA sequences were visualized and designed using SnapGene (version 4.3).

- RNA-seq data analysis: RNA-seq library sequence quality in demultiplexed fastq files was checked by FastQC (v0.11.5) and the low-quality reads and adaptor sequences were removed by Trim Galore (v0.4.1) using the default parameters. The pre-processed RNA-seq reads were mapped to the human reference genome (UCSC GRCh38/hg38) using STAR (2.6.0a) (--outFilterMismatchNoverLmax 0.05 --outMultimapperOrder Random --winAnchorMultimapNmax 100 --outFilterMultimapNmax 100) guided by the ENSEMBL (Release 87) gene models. Read counts per gene were extracted using TEtranscripts and normalised by DEseq2 (v1.16.1) in R. Differential

expression analysis was also performed using DEseq2 (v1.16.1). The resulting gene expression table was used for downstream analyses in Microsoft Excel (version 16.29) and R (version 3.2.3). Pearson's correlation analysis was performed using the R cor command. Unsupervised hierarchical clustering was performed using the R hclust function with (1-Pearson's correlation coefficient) as distance measures. The R prcomp function was used for Principal component analysis (PCA). Gene set enrichment analysis was performed using the GSEA software (v4.0.3) by the Broad Institute. Gene Ontology (GO) analysis was performed using DAVID (v6.8).

Venn diagrams were plotted using VennPainter (v1.2.0) and p-values for two overlapping datasets were calculated using a generalised hypergeometric test for multiple samples. For the co-expression analysis of differentially expressed genes, the gene-based Pearson's correlation coefficients were calculated for PRDM14-AID-Venus competent hESC and hPGCLC samples using R (version 3.2.3). All pairwise correlations r < 0.8 between genes were removed. The matrix was imported as an adjacency matrix into the R igraph package, and the Louvain method for community detection was performed on the resulting graph. For comparison with microarray data, the published data was normalised, log2-transformed and the differential expression was evaluated using the R limma package.

ChIP-seq analysis: The library sequence quality in demultiplexed fastq files was checked by FastQC (v0.11.5) and the low-quality reads and adaptor sequences were removed by Trim Galore (v0.4.1) using the default parameters. The trimmed ChIP-seq reads were aligned to the human reference genome (UCSC hg38) by the Burrows-Wheeler Aligner (0.7.15-r1142-dirty). Samtools (v1.3.1) was used to remove unmapped and low mapping quality reads (options: view-F 4 -q 20). Subsequently, duplicated reads and reads mapped to unlocalised contig (random), unplaced contigs (ChrUn) and blacklisted regions (obtained from http://mitra.stanford.edu/kundaje/akundaje/release/blacklists/hg38-human/) were removed using Samtools rmdup. This was followed by preliminary peak calling using macs2 (v2.1.2) callpeak against the corresponding inputs (options: -g 3e9 --keep-dup all). Percentage of reads in peaks was calculated using featureCounts and visualised with multiQC (v1.6.dev0). Before peakcalling, to compare the number of peaks between the three high-quality samples, the library size for all libraries was downsampled to 25 million reads. Peaks were then called again using macs2 (v2.1.2) callpeak against the corresponding inputs (options: -g 3e9 --keep-dup all --nomodel –extsize 157). The resultant peak files of the three samples were merged to generate a consensus set of 6486 peaks using bedtools (v2.26.0) merge (options: -d 100). The consensus peaks were processed and clustered by DeepTools (v3.0.2-1-ac19361) computeMatrix and plotHeatmap. Peak annotation, motif enrichment and differential peak analyses were performed by HOMER (v4.10.4). DeepTools (v3.0.2-1-ac19361)bamCoverage (options: -extendReads 157 --binSize 10 --normalizeUsing CPM --ignoreForNormalization chrX chrY) was used to generate bigwigfile for visualization in Integrative Genomics Viewer (IGV, v2.4.13). The integrated ChIP-seq and RNA-seq table was generated using R (version 3.2.3) to correlate changes in gene expression to PRDM14 binding. Venn diagrams were plotted using the R/Bioconductor (v3.5) ChIPseeker package (v1.12.1).

For manuscripts utilizing custom algorithms or software that are central to the research but not yet described in published literature, software must be made available to editors/reviewers. We strongly encourage code deposition in a community repository (e.g. GitHub). See the Nature Research guidelines for submitting code & software for further information.

Data

Policy information about availability of data

All manuscripts must include a data availability statement. This statement should provide the following information, where applicable:

- Accession codes, unique identifiers, or web links for publicly available datasets
- A list of figures that have associated raw data
- A description of any restrictions on data availability

High-throughput sequencing data has been deposited at the NCBI Gene Expression Omnibus (GEO) database. The general GEO accession number is https://www.ncbi.nlm.nih.gov/geo/query/acc.cgi?acc=GSE138675

- RNA-Seq: https://www.ncbi.nlm.nih.gov/geo/query/acc.cgi?acc=GSE138673
- ChIP-Seq: https://www.ncbi.nlm.nih.gov/geo/query/acc.cgi?acc=GSE138674

Field-specific reporting

Please select the one below that is the best fit for your research. If you are not sure, read the appropriate sections before making your selection.

☒ Life sciences ☐ Behavioural & social sciences ☐ Ecological, evolutionary & environmental sciences

For a reference copy of the document with all sections, see nature.com/documents/nr-reporting-summary-flat.pdf

Life sciences study design

All studies must disclose on these points even when the disclosure is negative.

Sample size	No statistical methods were used to predetermine sample size. The sample sizes were based on community standards for relevant experiments. The precise number sizes for each experiment are indicated in relevant figure legends and/or Methods.
Data exclusions	No data were excluded from the analysis.
Replication	All experiments presented in the manuscript were replicated multiple times (numbers of repeats are stated in the figure legends). All replication attempts were successful.

Randomization	The experiments were not randomized. Randomization was not necessary as all experiments included control groups +/- relevant treatments.
Blinding	The investigators were not blinded to allocation during experiments and outcome assessment, following the standard practices for the experiments performed. Blinding to allocation was not possible due to experimental design (the need for specific treatments for different samples groups). Whenever possible, automated and not manual analyses were performed (e.g. counting of positive and negative cells based on IF images was performed using a Fiji plugin).

Reporting for specific materials, systems and methods

We require information from authors about some types of materials, experimental systems and methods used in many studies. Here, indicate whether each material, system or method listed is relevant to your study. If you are not sure if a list item applies to your research, read the appropriate section before selecting a response.

Materials & experimental systems

n/a	Involved in the study
☐	☒ Antibodies
☐	☒ Eukaryotic cell lines
☒	☐ Palaeontology
☒	☐ Animals and other organisms
☐	☒ Human research participants
☒	☐ Clinical data

Methods

n/a	Involved in the study
☐	☒ ChIP-seq
☐	☒ Flow cytometry
☒	☐ MRI-based neuroimaging

Antibodies

Antibodies used

All antibodies used in the study are described in Supplementary Table 3, replicated as text below:

1) Alkaline Phosphatase PerCP-Cy5.5 #561508; used for Flow cytometry (0.5 µl/6 EBs); BD Pharmingen; validation: Irie N et al. 2015: FACS purification of human PGCLCs
2) Alkaline Phosphatase AF647 #561500; used for Flow cytometry (0.5 µl/6 EBs); BD Pharmingen; validation: Irie N et al. 2015: FACS purification of human PGCLCs
3) CD38 PerCP-Cy5.5 #303522; used for Flow cytometry (0.5 µl/6 EBs); BioLegend; validation: Irie N et al. 2015: FACS purification of human PGCLCs
4) CD38 AF647 #303514; used for Flow cytometry (0.5 µl/6 EBs); BioLegend; validation: Irie N et al. 2015: FACS purification of human PGCLCs
5) Alkaline Phosphatase AF488 #561495; used for Flow cytometry (5 µl/genital ridge); BD Pharmingen; validation: Tang WW et al. 2015: FACS purification of human PGCs
6) cKIT (CD117) PerCP-Cy5.5 #333950; used for Flow cytometry (25 µl/genital ridge); BD Pharmingen; validation: Tang WW et al. 2015: FACS purification of human PGCs
7) PRDM14 AB4350; used for ChIP and IF (1:100 for IF; 3 µl / 2.5 million cells in ChIP); Millipore; validation: IF: Irie N et al. 2015: IF on human PGCLCs; ChIP: antibody was validated in this manuscript by ChIP qPCR on PRDM14-target and control regions (Chia et al. 2010) in wild type and PRDM14-depleted hESCs (Supplementary Fig.7a).
8) OCT4 #611203; used for IF (1:200); BD Biosciences; validation: Tang WW et al. 2015: IF on human PGCs
9) SOX17 AF1924; used for IF (1:500); R&D Systems ; validation: Tang WW et al. 2015: IF on human PGCs
10) GFP ab13970; used for IF (1:1000); Abcam; validation: Manufacturer validated by IF on GFP-transfected NIH/3T3 cells
11) PRDM14 ABD121; used for ChIP (3 µl / 2.5 million cells); Millipore; validation: Antibody was validated in this manuscript by ChIP qPCR on PRDM14-target and control regions (Chia et al. 2010) in wild type and PRDM14 depleted hESCs (Supplementary Fig.7A)
12) GFP ab290; used for ChIP (0.5 µl / 2.5 million cells); Abcam; validation: Antibody was validated in this manuscript by ChIP qPCR on PRDM14-target and control regions (Chia et al. 2010) in wild type and PRDM14 depleted hESCs (Supplementary Fig.7A).
13) TFAP2C sc-8977; used for IF (1:100); Santa Cruz; validation: Tang WW et al. 2015: IF on human PGCs
14) BLIMP1 #14-5963; used for IF (1:50); eBioscience; validation: Tang WW et al. 2015: IF on human PGCs
15) SOX2 sc-17320; used for IF (1:200); Santa Cruz; validation: Irie N et al. 2015: IF on human PGCLCs
16) myc-tag #2276; used for IF (1:4000); CST; validation: Manufacturer validated by IF on COS cells transfected with a Myc-tagged protein
17) HA-tag #3724; used for IF (1:200); CST; validation: Manufacturer validated by IF on COS cells, transfected with an HA-tagged protein
18) V5-tag ab27671; used for IF (1:100); Abcam; validation: Manufacturer validated by IF on CHO cells overexpressing a V5-tagged protein
19) Ki-67 ab16667; used for IF (1:500); Abcam; validation: Irie N et al. 2015: IF on human PGCLCs
20) NANOG ab80892; used for IF (1:100); Abcam; validation: Manufacturer validated by IF on mES cells
21) UHRF1 #61342; used for IF (1:500); Active Motif; validation: Tang WW et al. 2015: IF on human PGCs
22) Alexa fluorophore (AF-488, 568 and/or 647)-conjugated secondary antibodies; used for IF (1:500); Invitrogen (ThermoFisher); validation: Irie N et al. 2015: IF on human ESCs, PGCs and PGCLCs

Validation	Validation is described individually for each antibody in the "Antibodies used" field above.

Eukaryotic cell lines

Policy information about cell lines

Cell line source(s)	N3-tdTomato hESC line had been derived in the lab as described in Kobayashi et al., Nature 2017.<br>H9 hESC line (Thompson et al., Science, 1998) was obtained from WiCell.<br>All other hESC lines described in the study were obtained by genetic manipulation of the above.<br>HEK-293T cells were obtained by Dr. Ran Brosh from S.A. Aaronson's lab at ISMMS and described in Brosh et al., 2016.<br>CF1 MEFs were purchased from GlobalStem. DR4 (drug-resistant) MEFs were purchased from WT-MRC Cambridge Stem Cell Institute.
Authentication	None of the cell lines used were authenticated.
Mycoplasma contamination	The initial cell lines used (N3-tdTomato and H9), as well as PRDM14-AID (Par) cell line tested negative for Mycoplasma contamination. The cell lines derived therefrom were not directly tested for Mycoplasma contamination, however RNA-seq was examined for Mycoplasma reads, and no contamination was found.
Commonly misidentified lines<br>(See ICLAC register)	None of the misidentified cell lines were used.

Human research participants

Policy information about studies involving human research participants

Population characteristics	Human fetal gonadal tissue (Wk7, Wk8, Wk9 female and Wk7, Wk8, Wk9 male gonads). No information is available about the mothers.
Recruitment	Fetal samples were collected from mothers who were over 18 years old and had already consented to termination of pregnancy at Addenbrooke's hospital. No genetic screening was performed, but gonads were of normal morphology.
Ethics oversight	The collection and usage of human embryonic tissues were approved by the National Research Ethics Service (NRES) Committee East of England - Cambridge Central, United Kingdom (REC 96/085).

Note that full information on the approval of the study protocol must also be provided in the manuscript.

ChIP-seq

Data deposition

[x] Confirm that both raw and final processed data have been deposited in a public database such as GEO.

[x] Confirm that you have deposited or provided access to graph files (e.g. BED files) for the called peaks.

Data access links<br>*May remain private before publication.*	https://www.ncbi.nlm.nih.gov/geo/query/acc.cgi?acc=GSE138674
Files in database submission	- auxin-treated hESCs: GSM4115964 GFP_ESC_4i_IAA_cl11 (NARROWPEAK provided)<br>- untreated hESCs: GSM4115965 GFP_ESC_4i_noIAA_cl11 (NARROWPEAK provided)<br>- untreated hPGCLCs: GSM4115966 GFP_PGCLC_noIAA_r2 (NARROWPEAK provided)<br>- input hESCs: GSM4115967 Input_ESC_4i<br>- input hPGCLCs: GSM4115968 Input_PGCLC
Genome browser session<br>(e.g. UCSC)	no longer applicable

Methodology

Replicates	The following samples were included in the analysis (one replicate per condition): 4i hESCs (cl11 no IAA), 4i+IAA hESCs (cl11 +IAA) and hPGCLCs (cl11 and cl21 pooled; no IAA).
Sequencing depth	All reads were obtained by single-end, 50 bp sequencing on HiSeq 4000 sequencing system (Illumina)<br><br>- auxin-treated hESCs: Total reads – 32713260, Assigned reads – 130513<br>- untreated hESCs: Total reads – 26142849, Assigned reads – 200769<br>- untreated hPGCLCs: Total reads – 30837912, Assigned reads – 302381<br>- input hESCs: Total reads – 31923784, Assigned reads – 37708<br>- input hPGCLCs: Total reads – 30342268, Assigned reads – 39497

Before peak calling the library size for all libraries was downsampled to 25 million reads.

Antibodies	Anti-GFP ChIP-grade antibody from abcam (ab290)
Peak calling parameters	Peaks were called using macs2 (v2.1.2) callpeak against the corresponding inputs (options: -g 3e9 --keep-dup all --nomodel –extsize 157).
Data quality	The library sequence quality in demultiplexed fastq files was checked by FastQC (v0.11.5) and the low-quality reads and adaptor sequences were removed by Trim Galore (v0.4.1) using the default parameters. The trimmed ChIP-seq reads were aligned to the human reference genome (UCSC hg38) by the Burrows-Wheeler Aligner (0.7.15-r1142-dirty). Samtools (version: 1.3.1) was used to remove unmapped and low mapping quality reads (options: view-F 4 -q 20). Subsequently, duplicated reads and reads mapped to unlocalised contig (random), unplaced contigs (ChrUn) and blacklisted regions (obtained from http://mitra.stanford.edu/kundaje/akundaje/release/blacklists/hg38-human/) were removed using Samtools rmdup. This was followed by preliminary peak calling using macs2 (v2.1.2) callpeak against the corresponding inputs (options: -g 3e9 --keep-dup all). Percentage of reads in peaks was calculated using featureCounts and visualised with multiQC. Before peakcalling, to compare the number of peaks between the three high-quality samples, the library size for all libraries was downsampled to 25 million reads. Peaks were then called again using macs2 (v2.1.2) callpeak against the corresponding inputs (options: -g 3e9 --keep-dup all --nomodel –extsize 157). The resultant peak files of the three samples were merged to generate a consensus set of 6486 peaks using bedtools (v2.26.0) merge (options: -d 100). Narrowpeak has the macs2 (v2.1.2) default qvalue cut off of 0.05Also see "Software and code" section above and the Materials and Methods in the manuscript.
Software	See "Software and code" section above and the Methods section in the manuscript.

Flow Cytometry

Plots

Confirm that:

- [x] The axis labels state the marker and fluorochrome used (e.g. CD4-FITC).
- [x] The axis scales are clearly visible. Include numbers along axes only for bottom left plot of group (a 'group' is an analysis of identical markers).
- [x] All plots are contour plots with outliers or pseudocolor plots.
- [x] A numerical value for number of cells or percentage (with statistics) is provided.

Methodology

Sample preparation	At least 6 embryoid bodies were washed in PBS and dissociated with 0.25% Trypsin-EDTA for 10 min at 37°C. Cells were washed, resuspended in FACS buffer (3% FBS in PBS) and incubated with anti-AP and anti-CD38 antibodies for 30-60 minutes at 4°C in the dark. After washing, the cells were resuspended in FACS buffer with 0.1 µg/ml DAPI and filtered through a 50 µm cell strainer.
Instrument	Flow cytometry was done using BD LSR Fortessa, while FACS was performed with Sony SH100 Cell Sorter.
Software	Data were analysed using FlowJo (version 10.0.7) from Tree Star.
Cell population abundance	For flow cytometry, typically 10,000 (and no less than 3,000) "live cell" (DAPI-negative) events were recorded. For RNA-seq, 10,000 AP+ 4i hESCs or 10,000 NANOS3-tdTomato+AP+ hPGCLCs (with the exception of hPGCLC cl21 replicate, where 3,000 cells were used) were sorted.
Gating strategy	We have added a figure (Supplementary Figure 10) exemplifying the gating strategy. The gating was performed as follows: the initial cell population was defined as the largest compact events cluster on the SSC-A vs FSC-A plot; then cell doublets were eliminated using FSC-A vs FSC-H plot; afterwards dead cells were eliminated based on DAPI-positivity; finally, gating on live (DAPI-negative) cells, hPGCLCs were defined as NANOS3-tdTomato+ AP+ (or NANOS3-tdTomato+ CD38+) cells. The boundaries between positive and negative gates were defined using unstained samples and negative controls (such as differentiation without cytokines).

- [x] Tick this box to confirm that a figure exemplifying the gating strategy is provided in the Supplementary Information.
