# Supplementary material for: A critical role of PRDM14 in human primordial germ cell fate revealed by inducible degrons
Source: Nat Commun. 2020 Mar 9;11:1282. doi: 10.1038/s41467-020-15042-0 (PMC7062732; doi:10.1038/s41467-020-15042-0)
Supplement: Supplementary file 4 — Description of Additional Supplementary Files [file 41467_2020_15042_MOESM4_ESM.pdf]

Description of Additional Supplementary Files

File Name: Supplementary Data 1
Description: RNA-seq data for sorted NANOS3-tdTomato^{+}AP^{+} hPGCLCs and AP^{+} hESCs. 2 auxin-sensitive clones (cl11 and cl21), as well as the parental control lacking TIR1 were differentiated with or without IAA for 4 days. For hESCs, the same cell lines were grown for 1 passage (3 days) with or without IAA.

File Name: Supplementary Data 2
Description: Comparative differential expression analysis of PRDM14 depletion in hPGCLCs (from Supplementary Data 1) and *TFAP2C*$^{-/-}$ or *PRDM1*$^{-/-}$ hPGCLCs (Kojima et al., 2017; Tang et al., 2015). RNA-seq data were re-normalised together to obtain an integrated gene expression table.

File Name: Supplementary Data 3
Description: ChIP-seq data for hPGCLCs and hESCs. 2 PRDM14-AID-Venus clones (cl11 and cl21), as well as the parental control cell line were used. Immunoprecipitation was carried out using the ChIP-grade anti-GFP antibody validated in Supplementary Figure 7A.

File Name: Supplementary Data 4
Description: Comparative differential expression analysis of PRDM14 depletion in hPGCLCs and hESCs (from Supplementary Data 1) and the transcriptome of mouse *Prdm14*$^{-/-}$ cells (mPGCLCs and mESCs) from Shirane et al., 2016. RNA-seq data were re-normalised together to obtain an integrated gene expression table.

File Name: Supplementary Data 5
Description: Oligos used in the study.

File Name: Supplementary Data 6
Description: Gene ontology (GO) analysis on hPGCLC RNA-seq from Supplementary Data 1.
